# Probabilistic Risk Assessment – The Keystone for the Future of Toxicology

**DOI:** 10.14573/altex.2201081

**Published:** 2022

**Authors:** Alexandra Maertens, Emily Golden, Thomas H. Luechtefeld, Sebastian Hoffmann, Katya Tsaioun, Thomas Hartung

**Affiliations:** 1 Center for Alternatives to Animal Testing (CAAT), Johns Hopkins University, Bloomberg School of Public Health, Baltimore, MD, USA;; 2 ToxTrack, Baltimore, MD, USA;; 3 seh consulting + services, Paderborn, Germany;; 4 CAAT Europe, University of Konstanz, Konstanz, Germany

## Abstract

Safety sciences must cope with uncertainty of models and results as well as information gaps. Acknowledging this uncertainty necessitates embracing probabilities and accepting the remaining risk. Every toxicological tool delivers only probable results. Traditionally, this is taken into account by using uncertainty / assessment factors and worst-case / precautionary approaches and thresholds. Probabilistic methods and Bayesian approaches seek to characterize these uncertainties and promise to support better risk assessment and, thereby, improve risk management decisions. Actual assessments of uncertainty can be more realistic than worst-case scenarios and may allow less conservative safety margins. Most importantly, as soon as we agree on uncertainty, this defines room for improvement and allows a transition from traditional to new approach methods as an engineering exercise. The objective nature of these mathematical tools allows to assign each methodology its fair place in evidence integration, whether in the context of risk assessment, systematic reviews, or in the definition of an integrated testing strategy (ITS) / defined approach (DA) / integrated approach to testing and assessment (IATA). This article gives an overview of methods for probabilistic risk assessment and their application for exposure assessment, physiologically-based kinetic modelling, probability of hazard assessment (based on quantitative and read-across based structure-activity relationships, and mechanistic alerts from *in vitro* studies), individual susceptibility assessment, and evidence integration. Additional aspects are opportunities for uncertainty analysis of adverse outcome pathways and their relation to thresholds of toxicological concern. In conclusion, probabilistic risk assessment will be key for constructing a new toxicology paradigm – probably!

## 1 Introduction

Nothing is as certain as death and taxes^1^. Toxicology (as all of medicine) does not reach this level of certainty, as the Johns Hopkins scholar William Osler (1849–1919) rightly stated, “*Medicine is a science of uncertainty and an art of probability*”, and in this sense toxicology is a very medical discipline. However, our expectation as to the outcome of safety sciences is certainty – a product coming to the market must be safe. This article aims to make the case that we are actually working with an astonishing level of uncertainty in our assessments, which we hide by using apparently deterministic expressions of results (classifications, labels, thresholds, etc.). It is not that we cannot know, but that our predictions have only a certain probability of being correct – not very comforting when the safety of sometimes millions of patients and consumers is at stake.

The 2017 book *The Illusion of Risk Control – What Does it Take to Live with Uncertainty?* edited by Gilles Motet and Corinne Bieder, makes the important point of acknowledging that there is always a risk and that we can only assess and manage its probability. Consequently, safety is defined by the absence of unacceptable risk, not as the absence of all risk. Giving up on the illusion of safety and acknowledging uncertainty does give a new perspective on risk assessment and management as we will discuss here, applying it to toxicology. Dupuy (1982) described the problem as “*The fundamental incapacity of Industrial Man to control his destiny increasingly appears as the paradoxical and tragic result of a desire for total control – either by reason or by force*”. As we will see, embracing uncertainty can free us to adopt a new toxicity testing paradigm.

*Uncertainty and probability* are two sides of the same coin. Risk assessment under uncertainty, therefore, logically leads us to probabilistic risk assessment (ProbRA). We will go light on mathematics here. This article is primarily about why to use ProbRA and not on how to do it. In recent years, the importance of having a firm understanding of probability has become apparent, and as a result there are several books the reader can consult, which we recommend:

- Kurt, Will (2019). *Bayesian Statistics the Fun Way*.
- Mlodinow, Leonard (2008). *The Drunkard’s Walk: How Randomness Rules Our Lives*.
- Wheelan, Charles (2013). *Naked Statistics: Stripping the Dread from the Data*.

## 2 Some defining characteristics of (un)certainty versus probability versus risk

### 2.1 Uncertainty

“*We know accurately only when we know little; with knowledge, doubt increases*” (Johann Wolfgang von Goethe in *Maxims and Reflections*).

**Fig. 1:**
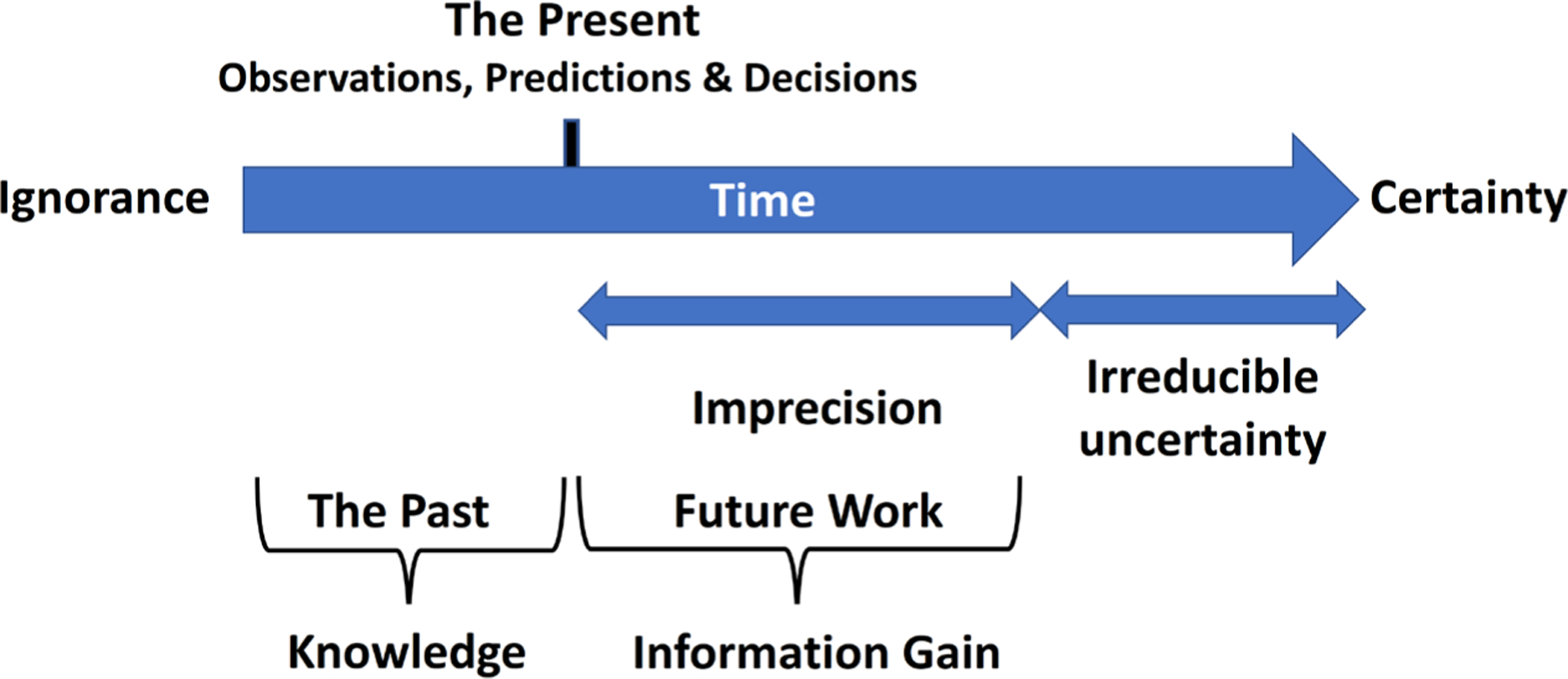
Knowledge gain versus uncertainty Modified and combined from Njå et al. (2017) and Augenbaugh (2006)

Uncertainty in toxicology is at its base the lack of knowledge of the true value of a quantity or relationships among quantities. Figure 1 illustrates the path from ignorance approximating certainty with some irreducible uncertainty remaining. Walker et al. (2003) note that uncertainty is not simply the absence of knowledge, but a situation of inadequate information (inexactness, unreliability, and sometimes ignorance). “*However, uncertainty can prevail in situations where a lot of information is available* …*. Furthermore, new information can either decrease or increase uncertainty. New knowledge on complex processes may reveal the presence of uncertainties that were previously unknown or were understated. In this way, more knowledge illuminates that our understanding is more limited or that the processes are more complex than thought before*”. Cullen and Frey (1999) address uncertainties that arise during risk analyses:

1. *Scenario uncertainty* – typically of omission, resulting from incorrect or incomplete specification of the risk scenario to be evaluated. In toxicology, for example, risk assessment before the actual use of a substance is clear.
2. *Model uncertainty* – limitations in the mathematical models or techniques often due to (a) simplifying assumptions; (b) exclusion of relevant processes; (c) misspecification of model boundary conditions (e.g., the range of input parameters); or (d) misapplication of a model developed for other purposes. In toxicology, this obviously resonates with many aspects of the risk assessment process.
3. *Input or parameter uncertainty* – particular attention must be paid to measurement error, which can be either systemic (when there is a bias in the data) or random (noise in the data). Toxicology obviously faces both, but these are rarely explicitly addressed when risk assessments are made.

Today, additional aspects such as inconsistency, bias, and methodological choices are considered as sources of uncertainty. Recent European Food Safety Authority (EFSA) guidance (EFSA, 2018) details uncertainty very comprehensively for the safety sciences.

The Grading of Recommendations, Assessment, Development and Evaluation (GRADE) working group has issued a guideline (Brozek et al., 2021) on assessing the certainty in modelled evidence, which includes the three types of uncertainty mentioned above and provides a flowchart for finding, selecting, and assessing certainty in a model. The certainty of modelled outputs is recommended to be assessed on the following domains:

1. Risk of bias
  - credibility of the model itself
  - certainty of all inputs
2. Directness
3. Precision
4. Consistency
5. Risk of publication bias

*Variability* (a.k.a. *imprecision*) refers to real differences in results over time, space, or members of a population and is a property of the system being studied (e.g., body weight, food consumption, age, etc. for humans or ecological species). Uncertainty is usually seen as the enemy of safety. But as Pariès (2017) rightly states, “*Uncertainty is not necessarily bad. Actually we are immerged in uncertainty, we live with it, and we need it to deal with the world’s complexity with our limited resources. We have inherited cognitive and social tools to manage it and deal with the associated unexpected variability. We need to better understand these tools and augment their efficiency in order to engineer resilience into our socio-technical systems*”.

### 2.2 Probability

Here we come to the core of the argument. Stephen Jay Gould (1941–2002, US paleontologist and historian of science) wrote in The Dinosaur in the Haystack (1995), “*Misunderstanding of probability may be the greatest of all impediments to scientific literacy*”. So, what is probability? George Boole (1815–1864, English mathematician and philosopher best known for his Boolean algebra) stated, “*Probability is expectation founded upon partial knowledge. A perfect acquaintance with all the circumstances affecting the occurrence of an event would change expectation into certainty, and leave neither room nor demand for a theory of probabilities*”. A probabilistic approach is based on the theory of probability and the fact that randomness plays a role in prediction. It is the opposite of deterministic. A *deterministic* situation, i.e., one without uncertainty, though does not exclude *imprecision* affecting our determination. Probabilistic models incorporate random variables and probability distributions into the respective model.

Few probabilities are known, like rolling a perfect die; they are called *a priori probabilities*. Where observed frequencies are used to predict probabilities, we call them *statistical probabilities*, to be distinguished from *estimated probabilities*, which are based on judgement because of the associated uncertainty. Almost all risk decisions in risk assessment are based on a combination of the latter two. The critical question is the reliability of the probability estimate. The purpose of this article is to stress that there are methods to assess the remaining uncertainty and support managing the resulting risk.

The key point we must clarify is that we are not just talking about the p-value of our statistical significance tests when talking about probabilities in risk assessment. Aside the poor use of statistics in toxicology in general (Hartung, 2013), it will surprise many readers that our gold-standard significance test approach, which is increasingly used (Cristea and Ioannidis, 2018), is actually ill-suited for the questions we ask ([Bibr R40][Bibr R41])^2^: “*Biological understanding and previous research play little formal role in the interpretation of quantitative results. This phenomenon is manifest in the discussion sections of research articles and ultimately can affect the reliability of conclusions. The standard statistical approach has created this situation by promoting the illusion that conclusions can be produced with certain ‘error rates,’ without consideration of information from outside the experiment. This statistical approach, the key components of which are P values and hypothesis tests, is widely perceived as a mathematically coherent approach to inference*.” The articles discuss the resulting “*p value fallacy*”. P value fallacy in easy terms means “*while most physicians and many biomedical researchers think that a ‘P’ of 0.05 for a clinical trial means that there is only a 5% chance that the null hypothesis is true, that is not the case. Here is what ‘P = 0.05’ actually means: if many similar trials are performed testing the same novel hypothesis, and if the null hypothesis is true, then it (the null) will be falsely rejected in 5% of those trials. For any single trial, it doesn’t tell us much*”. Ioannidis (2008) shows the problem for a large number of observational epidemiological studies. Seeing the comparatively high standard of statistics in clinical trials and epidemiology, we are for larger parts of science reminded of Nassim Taleb (2007), “*They only knew enough math to be blinded by it*”.

It should be noted that an understanding of probability developed only slowly in science; Pierre-Simon Laplace classically defined the probability of an event as the number of outcomes favorable to the event divided by the total number of possible outcomes. So, the probability of throwing a six with a perfect die is 1 in 6. Laplace finalized the classical probability theory in the 19^th^ century, which started as early as the 16^th^ century (especially Pierre de Fermat and Blaise Pascal in the 17^th^ century) mainly from the analysis of games. Jacob Bernoulli expanded to the principle of indifference, taking into account that not all outcomes need to have the same probability, and others expanded it to continuous variables. In 1933, the Russian mathematician A. Kolmogorov (1903–1987) outlined an axiomatic approach that forms the basis for the modern theory defining probability based on the three suggested axioms.

In the 20^th^ century, frequentist statistics was developed and became the dominant statistical paradigm. It continues to be most popular in scientific articles (with p-values, confidence intervals, etc.). Frequentist statistics is about repeatability and gathering more data, and probability is the long-run frequency of repeatable experiments.

An alternative approach is “Bayesian inference” based on Bayes’ theorem, named after Thomas Bayes, an English statistician of the 18^th^ century. Here, probability essentially represents the degree of belief in something, probably closer to most people’s intuitive idea of probability.

We can thus distinguish three major forms of probability:

1. The classical or axiomatic (based on Kolmogorov’s axioms) probability
2. The experimental / empirical probability of an event is equal to the long-term frequency of the event’s occurrence when the same process is repeated many times (also termed frequentist statistics or frequentist inference)
3. Subjective probability as the degree of belief or logical support (updated using Bayes’ theorem)

One drawback of the frequentist approach that is addressed by Bayesian inference is the issue of false-positives, especially for rare events (Szucs and Ioannidis, 2017). We have repeatedly stressed this problem for toxicology, where most hazards occur at low frequencies (Hoffmann and Hartung, 2005). The other way around, “big data” is bringing the reverse challenge of overpowered studies, i.e., “*massive data sets expand the number of analyses that can be performed, and the multiplicity of possible analyses combines with lenient P value thresholds like 0.05 to generate vast potential for false positives*” (Ioannidis, 2019). Another drawback is that frequentists neglect that opinion plays a major role in both preclinical and clinical research; Bayesian statistics forces the contribution of opinion out into the open where it belongs.

### 2.3 Likelihood

The distinction between probability and likelihood, a.k.a. reverse probability, is fundamentally important^3^: “*Probability attaches to possible results; likelihood attaches to hypotheses*.” This brings us to Bayesian statistics, which consider our beliefs. “*Hypotheses, unlike results, are neither mutually exclusive nor exhaustive. … In data analysis, the ‘hypotheses’ are most often a possible value or a range of possible values for the mean of a distribution. … The set of hypotheses to which we attach likelihoods is limited by our capacity to dream them up. In practice, we can rarely be confident that we have imagined all the possible hypotheses. Our concern is to estimate the extent to which the experimental results affect the relative likelihood of the hypotheses we and others currently entertain. Because we generally do not entertain the full set of alternative hypotheses and because some are nested within others, the likelihoods that we attach to our hypotheses do not have any meaning in and of themselves; only the relative likelihoods – that is, the ratios of two likelihoods – have meaning. … This ratio, the relative likelihood ratio, is called the ‘Bayes Factor’*.”^3^

In toxicology, our hypothesis is usually not articulated, but fundamentally we assume that a substance is toxic or, alternatively, that it is non-toxic. This set of hypotheses is neither complete nor mutually exclusive: The substance could be beneficial or toxic for some people or under certain circumstances. Results, on the contrary, refer to the outcome of a specific experiment where associated probabilities are adequate.

### 2.4 Risk

Risk has in the context of toxicology first to be distinguished from hazard, which is not always easy, as many languages do not make this distinction. Hazard is a source of danger, e.g., a tiger, but it becomes a risk only with exposure, i.e., a possibility of loss or injury with a certain probability. The tiger in the cage is a hazard with negligible risk.

Risk is characterized by two quantities:

1. the magnitude (severity) of the possible adverse consequence(s), and
2. the likelihood (probability) of occurrence of each consequence.

Table 1 gives examples of risks with the different combinations of these two properties.

Kaplan and Garrick (1981) defined risk in the context of toxicology as “*risk is probability and consequences*”. So, it is about the severity of possible damage or, as former U.S. Environmental Protection Agency (EPA) Administrator William K. Reilly phrased it, “*Risk is a common metric that lets us distinguish the environmental heart attacks and broken bones from indigestion or bruises*”^4^. For toxicology, risk is typically defined for an individual or a population. The consequences (hazards) are typically quite clear, but we struggle with the probabilities. Taleb (2007) phrased it outside of toxicology, “*We generally take risks not out of bravado but out of ignorance and blindness to probability!*”

## 3 The lack of certainty in toxicology

For the reader of this series of articles, this argument is a common thread. Some favorites in brief: In Hartung (2013, Tab. 1) we list 25 reasons why animal models as the most common approach do not reflect humans and cite studies that 20% of drug candidates fail because of unpredicted toxicities, and after passing clinical trials ~8% are withdrawn from the market mostly because of unexpected side-effects. Major studies by consortia of the pharmaceutical industry showed that rodents predict 43% of side effects in humans (n = 150) (Olson et al., 2000) and for all species had a sensitivity of 48% and specificity of 84% (n = 182) (Monticello et al., 2017).

Animal tests cannot be more relevant for humans than they are reproducible for themselves – we showed that of 670 eye corrosive chemicals, a repeat study showed 70% to be corrosive, 20% to be mild, and 10% to have no effect (Luechtefeld et al., 2016a). For skin sensitization, the reproducibility of the guinea pig maximization test was 93% (n = 624) and of the local lymph node assay (LLNA) in mice 89% (n = 296) (Luechtefeld, 2016b). Others reported for the cancer bioassay 57% reproducibility (n = 121) (cited in Basketter et al., 2012 and Smirnova et al., 2018). In our largest analysis (Luechtefeld et al., 2018b), we showed for the six most used Organisation for Economic Co-operation and Development (OECD) guideline tests and 3,469 cases where a chemical was tested more than twice, an average sensitivity of 69% (accuracy 81%); this means that the toxic property is missed in one of three tests.

Obviously, we usually do not know how well animal studies predict human health effects. However, interspecies comparisons cited in the papers above and in Wang and Gray (2015) allow an estimate, as there is no reason to assume that any species predicts humans better than they predict each other. These are some examples:

- Skin sensitization (n = 403): 77% guinea pig versus mouse
- Carcinogenicity (n = 317): 57% rat versus mouse
- Reproductive toxicity (n = 167): ~61% rat versus rabbit versus mouse
- Repeat dose toxicity (n = 37): 75–80% rat versus mouse; 27–55% for organ prediction
- Repeat dose toxicity (n = 310): 68% rat versus mouse

In conclusion, toxicity tests in animals done according to OECD guidelines and under Good Laboratory Practice conditions are roughly 80% reproducible, and different lab animal species are concordant about 60% of the time. This quite impressively illustrates the uncertainty with which we operate. These are tests to estimate human safety!

For ecotoxicology, Hrovat et al. (2009) have shown an enormous variability of test results: For 44 compounds with at least 10 data entries in the ECOTOX database each, they analyzed 4,654 test reports and report variability exceeding several orders of magnitude (up to 8, i.e., one hundred million).

It is important to realize that failure to be realistic about uncertainty in toxicology has significant consequences: When a chemical is declared “safe” only to be determined years later to result in unexpected toxicity, this increases public skepticism about the ability of science to protect people (Maertens et al., 2021).

These reproducibility problems matter especially for the low-frequency events we study (Hoffmann and Hartung, 2005). The problem of rare events of big impact has been elegantly covered by Nassim Taleb (2007) in his popular book *The Black Swan – The Impact of the Highly Improbable*. Some pertinent quotes^5^ were cited earlier in this series (Bottini and Hartung, 2009). A few others are sprinkled into this article. Furthermore, the reader is referred to Taleb’s earlier book (2004) on randomness, where many of the same ideas are formulated in a less populistic way. With respect to certainty of our (animal) tools in toxicology, the most appropriate quote from Taleb (2007) is, “*In the absence of a feedback process you look at models and think that they confirm reality*”.

Recently, the Evidence-based Toxicology Collaboration (EBTC^6^) has tried a new approach to assessing certainty by evaluating rare toxicological events of drug-induced liver injury (DILI), which are poorly predicted by the mandated regulatory test battery. EBTC has put together a multi-stakeholder working group, which has searched for published evidence of DILI effects of drugs with DILI and no-DILI. The approach demonstrated that mechanistic tests reported in the U.S. EPA ToxCast database, and not the mandated regulatory animal tests, predicted rare DILI in humans (Dirven et al., 2021). This evidence-based approach has potential for broader application in toxicological methods validation.

## 4 Probabilistic risk assessment (ProbRA) 101

In the American system, 101 indicates an introductory course, often with no prerequisites. In this spirit, let’s summarize the principles and refer to the more comprehensive literature for details (Kirchsteiger, 1999; Jensen, 2002; Vose, 2008; Modarres, 2008; Vesely, 2011; Ostrom and Wilhelmsen, 2012).

The probabilistic approach is the most widely used method of uncertainty analysis used in mathematical models. ProbRA has emerged as an increasingly popular analysis tool, especially to evaluate risks associated with every aspect of a complex engineering project (e.g., facility, spacecraft, or nuclear power plant) from concept definition, through design, construction, and operation, to end of service and decommissioning. It has its origin in the aerospace industry before and during the Apollo space program. ProbRA is a systematic and comprehensive methodology, which has only rarely been applied to substance safety assessments. ProbRA usually answers three basic questions as summarized by Michael Stamatelatos, NASA Office of Safety and Mission Assurance^7^:

1. “What can go wrong with the studied technological entity, or what are the initiators or initiating events (undesirable starting events) that lead to adverse consequence(s)?
2. What and how severe are the potential detriments, or the adverse consequences that the technological entity may be eventually subjected to as a result of the occurrence of the initiator?
3. How likely to occur are these undesirable consequences, or what are their probabilities or frequencies?”

Quite obviously, these can be applied to toxicology, where the initiator is exposure, and the adverse / undesirable consequences are hazard manifestations. For the purpose of this article, question 3 is obviously key. However, we will include some thoughts below on applying an uncertainty concept to adverse outcome pathways (AOP), which can be seen as the toxicological mechanistic aspects of questions 1 & 2. Stamatelatos^7^ further suggests the methodologies listed in Table 2 to answer the three questions above.

For toxicology, the U.S. EPA pioneered ProbRA with the 1997 release of EPA’s “Policy for Use of Probabilistic Analysis in Risk Assessment”^8^. It states that “*probabilistic analysis techniques as Monte Carlo analysis, given adequate supporting data and credible assumptions, can be viable statistical tools for analyzing variability and uncertainty in risk assessments*”. Monte Carlo simulation (see, for example, textbooks by Melchers, 1999, and Madsen et al., 1986) is a technique that involves using random numbers and probabilities to solve problems. Originally, the EPA used “Monte Carlo method” essentially synonymously with ProbRA.

The modern Monte Carlo method / simulation was developed in the late 1940s by Stanislaw Ulam and John von Neumann in the nuclear weapons projects at the Los Alamos National Laboratory. It is based on the law of large numbers that a random variable can be approximated by taking the empirical mean of independent samples of the variable, where the input parameters are selected according to their respective probability distributions. This repeated random sampling to obtain numerical results uses randomness to solve problems that might be deterministic in principle. This way, it propagates variability or uncertainty of model input parameters and overcomes the uncertainty or variability in the underlying processes. For each combination of input parameters, the deterministic model is then solved, and model results are collected until the specified number of model iterations (shots) is completed. This results in a distribution of the output parameters, which is often parametrized using a Markov chain Monte Carlo (MCMC) sampler.

The Monte Carlo method, however, is just one of many methods for analyzing uncertainty propagation, where the goal is to determine how random variation, lack of knowledge, or error affects the sensitivity, performance, or reliability of the system that is being modeled. An alternative probabilistic methodology is the first- and second-order reliability method (FORM/SORM), a.k.a. Hasofer-Lind reliability index, a semi-probabilistic reliability analysis method devised to evaluate the reliability of a system. It estimates the sensitivity of the failure probability with respect to different input parameters. The method was suggested for ProbRA (Zhang, 2010).

Among the typically applied statistical techniques are (non-) parametric bootstrap methods. A parametric method assumes an underlying model (e.g., lognormal distribution); a non-parametric method only depends on the data points themselves. The term “bootstrap” is suggested to refer to the saying “*to pull oneself up by one’s bootstraps*” as a metaphor for bettering oneself by one’s own unaided efforts. As a statistical method, it belongs to the broader class of resampling methods. Bootstrapping assigns measures of accuracy (bias, variance, confidence intervals, prediction error, etc.) to sample estimates (Efron and Tibshirani, 1993; Davison and Hinkley, 1997). A great advantage of bootstrap is that it makes it easy to derive estimates of variability (standard errors) and confidence intervals for estimators of the distribution, such as percentile points, proportions, odds ratios, and correlation coefficients.

Similarly, maximum likelihood estimation^9^ can characterize uncertainty estimates at low sample sizes by estimating the parameters of an assumed probability distribution (Rossi, 2018). Alternatives are least squares regression or the generalized method of moments. Advantages and disadvantages of maximum likelihood estimation are^10^:

+ If the model is correctly assumed, the maximum likelihood estimator is the most efficient estimator. Efficiency is one measure of the quality of an estimator. An efficient estimator is one that has a small variance or mean squared error.
+ It provides a consistent but flexible approach that makes it suitable for a wide variety of applications, including cases where assumptions of other models are violated.
+ It results in unbiased estimates in larger samples.
- It relies on the assumption of a model and the derivation of the likelihood function, which is not always easy.
- Like other optimization problems, maximum likelihood estimation can be sensitive to the choice of starting values.
- Depending on the complexity of the likelihood function, the numerical estimation can be computationally expensive^11^.
- Estimates can be biased in small samples.

The Bayesian network (BN)^12,13^, also called Bayes network, belief network, belief net, decision net or causal network, introduced by Judea Pearl (1988), is a graphical formalism for representing joint probability distributions. Based on the fundamental work on the representation of and reasoning with probabilistic independence originated by the British statistician A. Philip Dawid in the 1970s, BN aim to model conditional dependence and, therefore causation, by representing conditional dependence by edges in a directed graph. Through these relationships, inference on the random variables in the graph is conducted by using weighing factors. Nodes represent variables (e.g., observable quantities, latent variables, unknown parameters or hypotheses). BN offer an intuitive and efficient way of representing sizable domains, making modeling of complex systems practical. BN provide a convenient and coherent way to represent uncertainty in models. BN have changed the way we think about probabilities.

These different mathematical tools have been employed to carry out probabilistic approaches in risk assessment. In 2014, the EPA published *Probabilistic Risk Assessment Methods and Case Studies* (EPA, 2014)^14^, describing ProbRA as “*analytical methodology used to incorporate information regarding uncertainty and/or variability into analyses to provide insight regarding the degree of certainty of a risk estimate and how the risk estimate varies among different members of an exposed population, including sensitive populations or lifestages*” applicable to both human health and ecological risk assessment. Two National Academy of Science reports influenced the report, namely, the National Research Council (NRC)’s report *Science and Decisions: Advancing Risk Assessment* (NRC, 2009) and *Environmental Decisions in the Face of Uncertainty* (IOM, 2013).

There are several comprehensive guides on how to actually do ProbRA (Jensen, 2002; Vose, 2008; Modarres, 2008; Vesely, 2011; Ostrom and Wilhelmsen, 2012). For our arguments, it suffices to say that in ProbRA at least one variable in the risk equation is defined as a probability distribution rather than a single number. However, the vision put forward is that more and more aspects of the risk equation should be seen as probability distributions that can be combined to estimate risk to an individual or, cumulatively, to a population. This is equally applicable to human health risk assessment and to the environment. The big questions are:

- Is the method sufficiently advanced for the different aspects of the chemical risk assessment context?
- What are the advantages and challenges?
- What does it take to make them acceptable for regulators and bring them to broader use?

Different stakeholders have embraced this new approach to different extents. EPA and EFSA are clearly at the forefront. EPA already in 1997 (!) started defining what makes ProbRA approaches acceptable to them (Box 1).

## 5 Software for ProbRA

Several free and commercial software packages are available for ProbRA (Tab. 3).

### 5.1 Freely available

US EPA has compiled a sizable list of freely available modeling tools for ProbRA, such as RIVM’s ConsExpo and MCRA, ILSI’s CARES, and EPA’s PROcEED, to name a few. The complete list, descriptions, and links to models can be found on US EPA ExpoBox Website^28^. RIVM’s MCRA model is a comprehensive probabilistic risk tool, while ConsExpo, DEEMS-FCID/Calendex, CARES, and SHEDS are probabilistic exposure modeling tools for various exposure scenarios (e.g., consumer products to dietary and residential exposures) (Young et al., 2012).

Probabilistic Reverse dOsimetry Estimating Exposure Distribution (PROcEED), developed by the US EPA, is used to perform probabilistic reverse dosimetry calculations. In essence, PROcEED estimates a probability distribution of exposure concentrations that would likely have produced the observed biomarker concentrations measured in a given population, using either a discretized Bayesian approach, or, when an exposure-biomarker relation is linear, a more straightforward exposure conversion factor approach.

iRisk is a web-based tool created by the FDA that assesses risk associated with microbial and chemical contaminants in food using a probabilistic approach. Users enter data for the various factors, such as food, hazard, dose-response, etc. to generate a prediction. Further, the model can evaluate the effectiveness of prevention and control measures; the results are presented as a population-based estimate of health burden.

mc2d is an R package for two-dimensional (or second-order) Monte-Carlo simulations to superimpose the uncertainty in the risk estimates stemming from parameter uncertainty^29^. In order to reflect the natural variability of a modeled risk, a Monte-Carlo simulation approach can model both the empirical distribution of the risk within the population and of distributions reflecting the variability of parameters across the population.

### 5.2 Commercial

Although not exhaustive, we outline some of the commercially available tools for ProbRA here. Agena Risk (Fenton and Neil, 2014) is a commercial software for Bayesian artificial intelligence (A.I.) and probabilistic reasoning for assessing risk and uncertainty in fields such as operational risk, actuarial analysis, intelligence analysis risk, systems safety and reliability, health risk, cyber-security risk, and strategic financial planning.

Oracle’s Crystal Ball and Palisade’s @Risk are commercially available applications used in spreadsheet-based tools to report and measure risk using Monte Carlo analysis. Advantages to these applications include multiple pre-defined distributions and the ability to use custom data distributions, which improves risk estimates. The user can also carry out a sensitivity analysis to identify the most impactful metrics.

### 5.3 PBPK / PBTK model software

Paini et al. (2017) summarized a number of PBK modeling software packages (Tab. 4), noting that “*the field as a whole has suffered from a fragmented software ecosystem, and the recent discontinuation of a widely used modelling software product (acslX) has highlighted the need for software tool resilience. Maintenance of, and access to, corporate knowledge and legacy work conducted with discontinued commercial software is highly problematic. The availability of a robust, free to use, global community-supported application should offer such resilience and help increase confidence in mathematical modelling approaches required by the regulatory community*”.

## 6 Probability of exposure

The concept that exposure has a certain probability for an individual and cumulatively for the population is intuitive and broadly used (Bogen et al., 2009). Cullen and Frey (1999) wrote a textbook, *Probabilistic Techniques in Exposure Assessment, on the concept*. Bogen et al. (2009) give a very comprehensive review on probabilistic exposure analysis for chemical risk characterization based on a Society of Toxicology’s Contemporary Concepts in Toxicology meeting (Probabilistic Risk Assessment (PRA): Bridging Components Along the Exposure-Dose-Response Continuum, held June 25–27, 2005, in Washington, DC). Jager et al. (2000) give a very comprehensive example for two substances, an existing chemical (dibutyl phthalate, DBP) and a new chemical notification (undisclosed) and present a review of the approach (summarized also in Jager et al., 2001). Chiu and Slob (2015) suggested a unified probabilistic framework for dose-response assessment of human health effects. EFSA in 2012 published extensive *Guidance on the Use of Probabilistic Methodology for Modelling Dietary Exposure to Pesticide Residues*^39^.

The German Federal Institute for Risk Assessment (BfR) lauds probabilistic exposure assessment^40^: “*Exposure assessment can help to determine the type, nature, frequency and intensity of contacts between the population and the contaminant that is to be assessed. Traditional exposure assessment (also called deterministic estimate or point estimate, ‘worst case estimates’) of risks from chemical substances estimates a value that ensures protection for most of the population. Deviations from the real values are tolerated in order to ensure protection of the consumer using simple methods by, in some cases, considerably overestimating actual exposure*.

For some time now the use of probabilistic approaches (also called distribution-based or population-related approaches) has been under discussion for exposure assessment. These methods do not merely describe a single, normally extreme case but rather endeavour to depict overall variability in the data and, by extension, to present all possible forms of exposure. The mathematical tools used in this approach are Monte Carlo simulations, distribution adjustments and other principles taken from the probability theory.

In toxicology risks are normally described by establishing limit values. Below a limit value there should be no risk; above a limit value health effects through contact with the chemicals cannot be ruled out. This approach is frequently challenged. The question has been raised whether this approach does justice to transparent, realistic risk assessment. Probabilistic methods could highlight this supposed lack of clarity, help to characterise uncertainties and take them into account in risk assessment.”

Exposure assessments are complex and have clearly limited throughput. They can typically target only a few substances, and individual exposures over time are highly diverse. Depending on the agent studied, either peak exposures or cumulative amounts are relevant. Metabolism of the chemical and interindividual differences add to the complexity. Noteworthy, approaches for rapid exposure assessment exist, such as US EPA’s ExpoCast project^41^, which allow triaging chemicals of irrelevant exposure (Wambaugh et al., 2015). Probabilistic approaches are again critical components here.

With the rise of biomonitoring studies, internal exposures, especially blood and tissue levels of chemicals, are increasingly becoming available. These depend on exposure and bioavailability (and other biokinetic properties to be discussed next). They offer opportunities to focus on relevant exposures. The concept has been broadened to exposomics (Sillé et al., 2020), which often employs probabilistic analyses for our context here.

## 7 Probability as the basis of PBPK / PBTK modeling

We have stressed earlier in this series and elsewhere the importance of pharmacokinetic modeling for modern toxicology (Basketter et al., 2012; Leist et al., 2014; Tsaioun et al., 2016; Hartung, 2017a, 2018a). Pharmacokinetic modeling plays a critical role in informing us whether a given dose of a chemical reaches a critical level at the target organ and, in reverse, what *in vitro* active concentrations correspond to as exposure needed, i.e., quantitative *in-vitro*-to-*in-vivo*-extrapolation (QIVIVE) (McNally et al., 2018).

Here, the most important message in the context of ProbRA is that the most advanced body of probabilistic methods is available as physiologically based pharmacokinetic / toxicokinetic (PBPK/PBTK) modeling (McLanahan et al., 2012). PK / TK theoretical foundation, practical application, and various software packages have been developed in pharmacology (Leung, 1991) and later adapted to toxicology (Bogen and Hall, 1989) for the environmental health context by friends and collaborators such as Mel Andersen, Bas Blaauboer, Frederic Bois, Harvey Clewell, George Loizou, Amin Rostami-Hodjegan, Andrew Worth and others; please see their work for more substantial discussions. Several workshops have documented the field (Tab. 5). Most recently, a textbook became available (Fisher et al., 2020). Loizou et al. (2008) stress the need for kinetics in risk assessment: “*The need for increasing incorporation of kinetic data in the current risk assessment paradigm is due to an increasing demand from risk assessors and regulators for higher precision of risk estimates, a greater understanding of uncertainty and variability …, more informed means of extrapolating across species, routes, doses and time …, the need for a more meaningful interpretation of biological monitoring data … and reduction in the reliance on animal testing … . Incorporating PBPK modelling into the risk assessment process can advance all of these objectives*.”

## 8 Probability of hazard

What indicates a probability of hazard? These four principal components come to mind:

1. Traditional test data on the given substance, which can range from physico-chemical measurements to animal guideline studies.
2. Such information on similar substances enabling (automated) read-across.
3. Structural alerts such as functional groups or chemical descriptors enabling (quantitative) structure-activity relationships ((Q)SAR).
4. Mechanistic alerts typically from *in vitro* testing or (clinical) biomarkers.

How these (jointly) indicate a probability of hazard and how to quantify it, is usually not clear. Some elements are more established. We have shown earlier how a combination of (1) and (2) can be used to derive probabilities of hazard (Luechtefeld et al., 2018a,b). These probabilities or, the other way around, measures of uncertainty are among the most remarkable features of the approach (Hartung, 2016) as they indicate whether more information is needed. The approach called read-across-based structure-activity relationship (RASAR) covers the nine most frequently used animal test-based classifications by OECD test guidelines. The method has been implemented as Underwriters Laboratories (UL) Cheminformatics Toolkit^42^; it has been further developed utilizing deep learning, making (non-validated) estimates of potency as GHS hazard classes and handling applicability domains of chemicals more explicitly. Notably, the method has been included in the new Australian chemicals legislation^43^, the Industrial Chemicals Act 2019 or AICIS (Australian Industrial Chemicals Introductions Scheme) in effect since July 1, 2020. This law creates a new regulatory scheme for the importation and manufacture of industrial chemicals by Australia. Unlike other jurisdictions, “industrial chemicals” includes personal care and cosmetics, and there is a full ban on new animal testing for these ingredients and dual-use ingredients that are used both in cosmetics and industrial uses. However, broader international acceptance of read-across as promoted also by the EUToxRisk project^44^ is still outstanding (Chesnut et al., 2018; Rovida et al., 2020). Other A.I.-based methods for hazard identification, which are more or less explicit in expressing probabilities of their predictions, are available (Zhang et al., 2018; Santin et al., 2021).

The approach under (3) is well-known as (Q)SAR, which has been covered earlier in this series of articles (Hartung and Hoffmann, 2009). (Q)SAR are based on structural alerts and physicochemical descriptors. Currently, we are exploring the integration of (Q)SAR as input parameters of the RASAR approach.

Most development is needed for (4). A read-across type of approach has been introduced for the US EPA ToxCast^45^ data (Shah et al., 2016), which tested about 2,000 chemicals in hundreds of robotized assays. This was also termed generalized read-across^46^. Pioneering work showed how to use this to predict endocrine activity (Browne et al., 2015; Kleinstreuer et al., 2018a; Judson et al., 2020). However, it is not clear how to extend this to chemicals that were not included in the ToxCast program. We discussed the opportunities of read-across of such biological data earlier (Zhu et al., 2016).

Most toxicologists, out of habit, talk of a xenobiotic exposure “causing” a certain effect, e.g., genotoxins cause cancer, etc. Yet, in reality, this is rarely the case – even when chemical exposures have a clear role in both initiation and progression, there is still a strong stochastic element involved (Tomasetti et al., 2017). For example, bilateral breast cancer is very rare, although both tissues have identical exposures. For other endpoints, it is even more important to remain mindful of the uncertainty intrinsic to most of the causal associations we are looking for in toxicology: For most diseases (Alzheimer’s and autism to name a few) we know that the environment plays an important role; however, decades of studies have failed to find any chemical “smoking gun”. We are instead likely looking for multiple exposures, over a lifetime, each of which may be individually insignificant, but which can, in vulnerable individuals, act as a tipping point.

One conceptual alternative to asking which chemicals “cause” which diseases is instead thinking of potential chemicals as quantifiable liabilities in a threshold-liability model. The threshold-liability model holds that for a given disease there exists within the population some probability distribution of thresholds, with some individuals with a high threshold (the life-long smoker who fails to develop lung cancer or heart disease) and others with considerably lower thresholds. Disease happens when an individual’s liabilities (which can include environmental exposures, stochastic factors, and epigenetic alterations) exceed their threshold. Such a model has been applied to amyotrophic lateral sclerosis (ALS) – a disease that has no known replicable environmental factors and is likely best characterized as the result of a pre-existing genetic load that faces environmental exposures over a lifespan and eventually reaches a tipping point, wherein neurodegeneration begins. While the past decade has seen an enormous expansion in our understanding of the genetic load component thanks to large-scale genome-wide association studies, the environmental component remains poorly characterized. While this is no doubt in part due to the much larger search space for environmental exposures, it must be acknowledged that the tools toxicologists employ – for example, looking for chemicals that will cause an ALS-like neurodegenerative phenotype in rodents at very high doses – are likely not ideal (Al-Chalabi and Hardiman, 2013).

An area where ProbRA has shown important (but largely neglected) opportunities is the test battery of genotoxicity assays. Depending on the field of use, three to six *in vitro* assays are carried out and, typically, any positive result is taken as an alert, leading to a tremendous rate of false-positive classifications as discussed earlier (Basketter et al., 2012). Aldenberg and Jaworska (2010) applied a BN to the dataset assembled by Kirkland et al., showing the potential of a probabilistic network to analyze such datasets. Expanding on work by Jaworska et al. (2013, 2015) for skin sensitization potency, we earlier showed how probabilistic hazard assessment by dose-response modeling can be done using BN (Luechtefeld et al., 2015). Our contribution was more technical (using feature elimination instead of QSAR, hidden Markov chains, etc.), but it moved the model’s potency predictions to standing cross-validation. Most recently, Zhao et al. (2021) compiled a human exposome database of > 20,000 chemicals, prioritized 13,441 chemicals based on probabilistic hazard quotient and 7,770 chemicals based on risk index, and provided a predicted biotransformation metabolite database of > 95,000 metabolites. While the importance of acute oral toxicity for ranking chemicals can be argued, it shows impressively how probabilistic approaches can be applied to large numbers of substances to allow prioritization.

## 9 Probability of risk

The prospect of ProbRA is increasingly recognized by regulators as shown earlier for EPA, EFSA and BfR (Tralau et al., 2015) and opinion leaders in the field (Krewski et al., 2014). A framework for performing probabilistic environmental risk assessment (PERA) was proposed (Verdonck et al., 2002, 2003). Risk assessment obviously requires combining hazard and exposure information; van der Voet and Slob (2007) suggested an approach where exposure assessment and hazard characterization are both included in a probabilistic way. Table 6 gives a few examples of ProbRA; notably they are very different in approach and quality, but they illustrate possible applications. Slob et al. (2014) used the ProbRA approach to explore uncertainties in cancer risk assessment. Together, this very incomplete list of examples of ProbRA in toxicology shows the potential of the technology.

## 10 Uncertainty and the adverse outcome pathway (AOP) concept

As discussed above, a key element of ProbRA is the analysis of how the system is challenged and can fail. This is reminiscent of the AOP approach, which can be seen as the implementation of the call for toxicity pathway mapping from the “Toxicity testing in the 21^st^ century movement” (Krewski et al., 2020). Based on the respective National Academy of Sciences / NRC report (NRC, 2007), a change toward new approach methodologies (NAMs) away from traditional animal testing, which is based on mechanistic understanding, i.e., toxicity pathways, pathways of toxicity (PoT) (Hartung and McBride, 2011; Kleensang et al., 2014) or, increasingly, AOP (Leist et al., 2017) is suggested.

A major obstacle to the introduction of NAMs in regulatory decision-making has been the lack of confidence, or substantial overall uncertainty, in their fitness-for-purpose. While some individual aspects of NAMs contributing uncertainty are assessed in a systematic and thorough manner, a comprehensive approach that maps all uncertainties involved is lacking. A generic framework that integrates current mechanistic knowledge, e.g., condensed into AOP, biological plausibility of NAMs in relation to that knowledge, and NAM reproducibility with well-established risk assessment-related uncertainties, such as intra- and interspecies differences, has the potential to provide a widely agreed basis for a realistic purpose-focused assessment of NAMs. For a given question, e.g., the determination of a specific health hazard, mapping available evidence for the various uncertainty sources onto the framework will provide a complete overview of strengths, weaknesses, and gaps in our mechanistic understanding and ask is the NAM relevant for the health effect? Such an understanding will not only guide future NAM development, but it also allows to uncouple current regulatory practices, i.e., essentially animal-based approaches, from the aim of assessing health effects in humans.

**Fig. 2:**
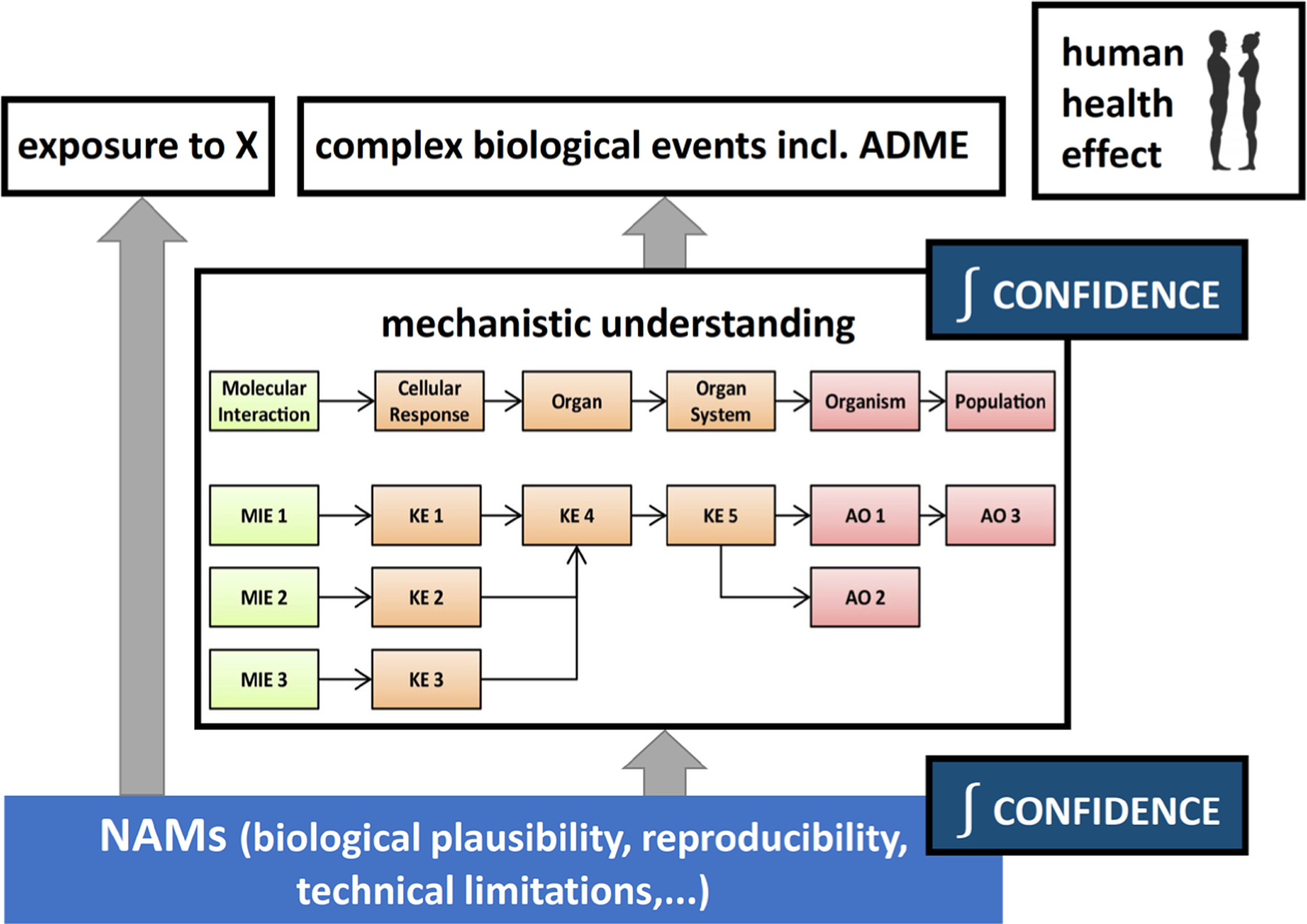
Increasing confidence in new approach methodologies (NAM) through mechanistic understanding and biokinetics of human health effects

Animal-based approaches are deeply rooted in regulatory approaches, but also in toxicology and environmental health, so that they are often used as a surrogate aim, not making their strengths and weaknesses explicit and transparent. A clear separation of the two would enable a fair and transparent assessment of NAMs, unbiased by current animal-based practices, for the purpose of protecting human health. Depending on the complexity of the human health effect, this approach will provide a clear path to reducing the overall uncertainty in NAM to achieve sufficient confidence in their results (Fig. 2).

For the identification of sources of uncertainty, uncertainty in our mechanistic understanding of the biological events that lead to human health effects needs to be identified by systematically mapping the peer-reviewed literature that has addressed this topic. Outcomes of recent workshops organized by the EBTC^6^ (de Vries et al., 2021; Tsaioun et al., in preparation), relevant information from national and international bodies, especially the guidance and case studies of the OECD, and the opinions of leading scientists should be incorporated. The sources of uncertainties in NAM need to be identified using a similar approach, with a focus on literature and other information on the assessment of individual NAM and combinations of NAM in testing strategies.

In order to build the generic framework, the literature can be screened for initiatives in the field of toxicology and environmental health that could be built upon, e.g., by Bogen and Spear (1987). A top-down approach is recommended that starts with a (close to) ideal situation: That is either the theoretical assumption that hazard or risk for a certain health effect upon exposure to an stressor X is known, i.e., quantifiable without uncertainty, or the more practical assumption of adapting the concept of a “target” trial, i.e., a hypothetical, not necessarily feasible or ethical trial, conducted on the population of interest, whose results would answer the question (see, e.g., Sterne et al., 2016). The aim of addressing a human health effect exclusively with NAM and identifying the uncertainties introduced by each step could be achieved by careful mapping of interdependence of sources of uncertainty and will be essential for their integration. This process needs to consider lessons learned from the deterministic and probabilistic integration of uncertainties of animal studies that can be transferred to NAM.

**Fig. 3:**
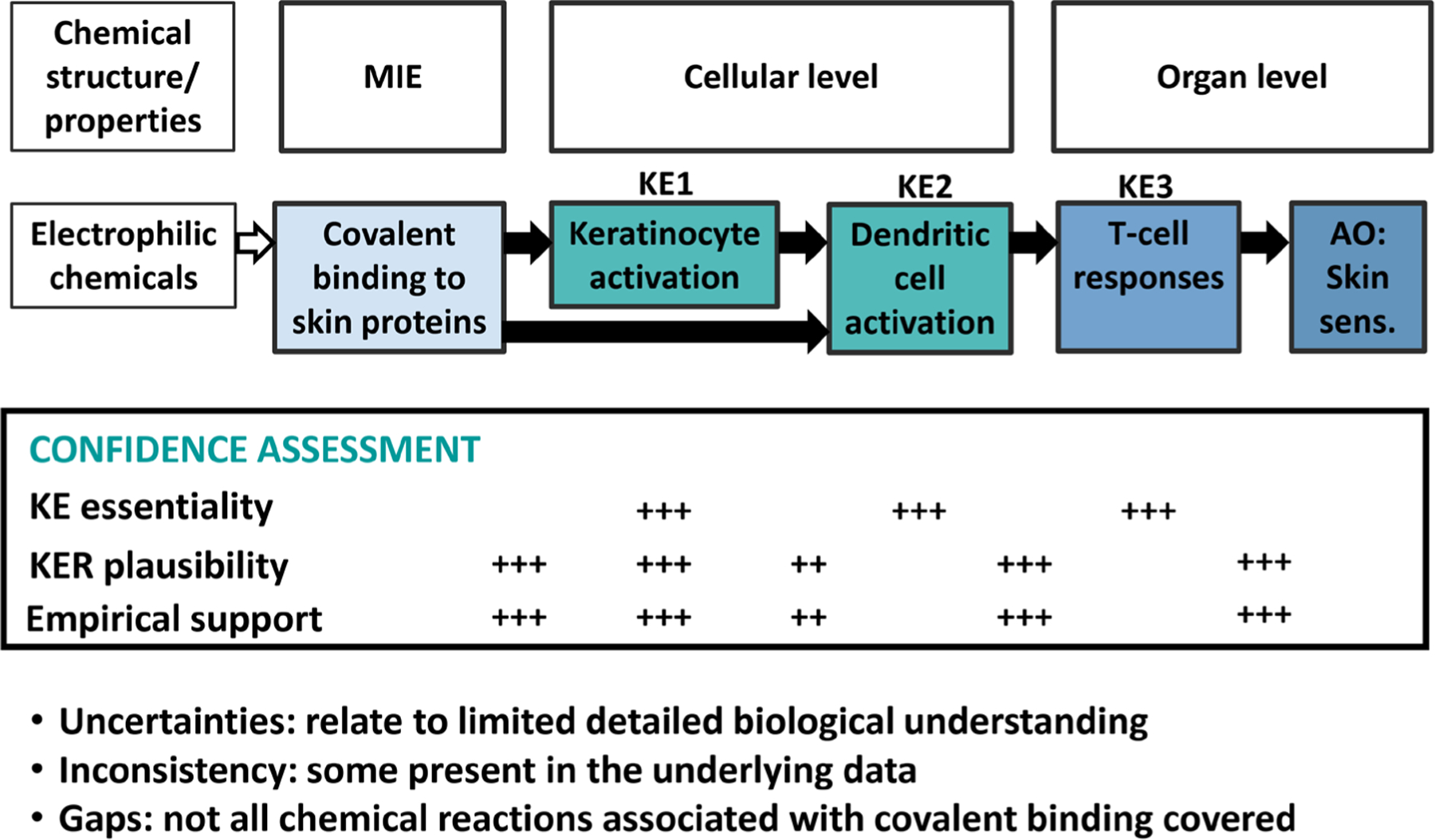
Example of skin sensitization adverse outcome pathway (AOP) confidence assessment MIE, molecular initiating event; KE, key event; AO, adverse outcome

The resulting frameworks could be explored by applying a select one as a case study. For illustration, skin sensitization hazard identification and risk assessment lends itself to this purpose for the following reasons:

- low complexity of the etiology of skin sensitization
- availability of a well-described AOP (Fig. 3), including formal confidence assessment^47^ (OECD 2014)
- availability of NAMs for the AOP events, many as OECD Test Guidelines (OECD 2018a,b, 2020)
- well-characterized NAMs, e.g., limitations, reproducibility, etc. (Hoffmann et al., 2018)
- availability of testing strategies, so-called defined approaches (DA) (Kleinstreuer et al., 2018b)
- next generation skin sensitization risk assessment (NGRA) approach of cosmetic ingredients (Gilmour et al., 2020)

Available evidence for the various sources of uncertainty needs to be collected and plugged into the framework. Interdependencies of uncertainties can be explored or modelled, where applicable, to inform a qualitative or semi-quantitative integration of all uncertainties to characterize the confidence in the final decision.

The main results would be a generic framework that maps all sources of uncertainty in NAM-based regulatory decisions on human health. Such an objective evidence-based framework enables a transparent fit-for-purpose assessment of NAM and NAM combinations, e.g., integrated approaches to testing and assessment (IATA) (OECD, 2017). Application of the framework will allow for mapping of NAMs and characterization of uncertainty in an integrative manner, while highlighting the strengths but especially the weaknesses and knowledge and NAM gaps. This in turn will help direct future research to address the identified shortcomings. Ultimately, such a comprehensive and transparent approach is a pre-requisite to increase the regulators’ confidence in NAM-based decision-making to a level that will allow abandoning the traditional animal-based approaches, not least as it allows comparison of the approaches.

## 11 Evidence-based medicine / toxicology and the role of probability and uncertainty

Rysavy (2013) titled an editorial “*Evidence-based medicine: A science of uncertainty and an art of probability*”. In fact, a lot of the change brought about by evidence-based medicine is replacing the eminence-based (authoritarian) black-and-white of “*this is the diagnosis/this is the treatment*” to an acceptance of uncertainties, probabilities for differential diagnoses, treatment options, and associated odds for outcome etc., exactly what we describe for ProbRA and its challenge to classification and labeling of toxicities. By promoting transparency and mapping uncertainties and biases as well as broad evidence use, ProbRA promotes very similar goals to evidence-based toxicology.

## 12 Thresholds of toxicological concern (TTC) as probabilistic approaches

TTC represent a bit of a hybrid between the two worlds. They are based on the distribution of no adverse effect levels (NOAEL), and then the 5^th^ percentile is used as a threshold, applying a safety factor of typically 100 (Hartung, 2017b). Future refinements of the concept might embrace uncertainty and probability considerations. As shown below, TTC might already now serve a role in the ProbRA approach.

## 13 Probabilistic avatars

Virtual representations of patients (avatars, digital twins)^48,49^ are increasingly developed as an approach to personalized medicine and even virtual clinical trials (Brown, 2016; Bruynseels et al., 2018). The European DISCIPULUS Project^50,51^ developed a roadmap for research and development. Earlier (Hartung, 2017c), we suggested that this is a logical extrapolation of the AOP concept: “*A virtual patient is not far from the creation of a personal avatar for each patient, where the standard model is adapted to the genetic and pharmacokinetic parameters of the patients and where interventions can be modeled and optimized in virtual treatments. Certainly still largely science fiction, but these were any of the technologies of our current toolbox some decades ago too*”. Here, it is important to note that the key underlying concept is the probabilistic approach of PB-PK. Similar to modeling disease and treatment, the hazardous consequences of exposure might be modelled in the future.

Noteworthy, this is also an interesting concept in the context of animal testing. Similar avatars of experimental animals might help with species extrapolations. Furthermore, we often point out that tests like the Draize rabbit eye test are not very reproducible. One source of variance is probably the animals themselves. Modeling the result of an animal test as a function of the chemical and animal tested (here avatar of the animal) would probably explain some of the uncertainty.

## 14 Artificial intelligence (A.I.) as the big evidence integrator delivers probabilities

A central problem of toxicology is evidence integration. More and more methodologies and results, some conflicting and others difficult to compare, are accumulating. We are facing this problem in more and more risk assessments, just thinking of tens of thousands of publications on bisphenol A, for example. Similarly, systematic reviews (Hoffmann et al., 2017; Farhat et al., 2022; Krewski et al., 2022) need to combine different evidence streams (EFSA and EBTC, 2018; Krewski et al., in preparation). Last but not least, the combination of tests and other assessment methods in integrated testing strategies (Hartung et al., 2013; Tollefsen et al., 2014; Rovida et al., 2015), a.k.a. IATA or DA by OECD, need to integrate different types of information. Again, probabilistic tools lend themselves to all of these.

We have earlier discussed how probabilistic approaches can help with integrated testing strategies, for example by determining the most valuable (next) test (Hartung et al., 2013). Briefly, we can ask how much the overall probability of the result can change with any outcome. Often, we might conclude that this is not actually worth the additional work, bringing an end to endless testing. Value of information analysis (Keisler et al., 2013) has enormous potential in toxicological decision-taking. This leads us to a type of *information economics*. Information economics is the discipline of modeling the role of information in an economic system as a fundamental force in every economic decision. We have stressed economic considerations earlier in this series of articles (Meigs et al., 2018). It seems like an interesting extension of this thinking if the investment into testing is contrasted quantitatively with the possible gain.

In the extreme, toxicology is seeing the rise of *big data*, which is defined by the three Vs: volume, velocity, and variety. These are key to understanding how we can measure big data and just how very different big data is to traditional data. Different technologies fuel this, such as omics technologies, high-content imaging, robotized testing (e.g., by ToxCast and the Tox21 alliance), sensor technologies, curated legacy databases, scientific and grey literature of the internet, etc. (Hartung and Tsatsakis, 2021). A.I. is making big sense from big data (Hartung, 2018b). It is worth mentioning that machine learning approaches frequently struggle with probabilities. Several existing approaches attempt to merge machine learning methods with probabilistic methods by modeling distributions or using Bayesian updating^52^. Frequently the outputs of neural networks are interpreted as probabilities, which can be problematic. Here, more work needs to be done.

Most importantly, by adopting a probabilistic view on safety information, we might come to a more flexible use of new approaches over time. If we do not see an individual method as definitive but only changing probabilities, we might be able to avoid the “war of faith” on the usefulness of animal tests, for example. Over time, we will see how the individual evidence sources contribute to the result of our A.I.-based integration. This might allow phasing out those methods that do not deliver valuable information and implementing those that do.

## 15 Conclusions and the way forward

As soon as we accept that risk assessment occurs with uncertainty and give up on the illusion of absolute safety, we must deal with probabilities. This is what science can deliver, as every experiment can only approximate truth. Working with models of reality with limited resources and technologies, and inherent variabilities and differences introduces uncertainty. The advantage of ProbRA is making these visible and estimating their potential contribution. By quantifying these uncertainties, we do not always need to default to the most conservative “precautionary” approach but can define acceptable risks and deprioritize scenarios clearly below them. ProbRA of chemicals offers numerous advantages compared to traditional deterministic approaches as well as several challenges^53^ (Tab. 7) (Kirchsteiger, 1999; Verdonck et al., 2002; Scheringer et al., 2002; Parkin and Morgan, 2006; Bogen et al., 2009; EPA, 2014).

The impressive list of advantages strongly encourages embracing the concept of ProbRA, especially as it makes more (transparent) use of evidence, something the authors have been arguing for in the context of evidence-based toxicology. This is reminiscent of “factfulness” as coined by Hans Rosling and coauthors (2018), who remind us in a very different context why we fail to recognize a changing world and grasp new insights. A major challenge is education, as the lack of familiarity among stakeholders and the public with ProbRA is a major challenge: “*Many view PRA [ProbRA] as a highly technical discipline that uses sophisticated mathematics and requires extensive training to apply and understand. Single point estimates are easier to grasp for most people, based in part on familiarity with this approach over the history of EPA. Although some people initially have difficulty interpreting probability distributions of values, everyone has a common baseline experience with probability, uncertainty and variability from everyday life (e.g., weather forecasting, odds of winning a lottery), and this experience could be used to frame the discussion of results. It is not necessary to understand the underlying mathematics or even to include results as full distributions. Results can be distilled down to the critical essence or decision-meaningful input of interest*.” (EPA, 2014). To contrast this optimistic view on communicating our scientific uncertainty, Bertrand Russell stated, “*The fundamental cause of the trouble is that in the modern world the stupid are cocksure while the intelligent are full of doubt*”.

Regulatory agencies play a key role for the implementation of ProbRA: The US EPA concluded in 2014 that “*Strategic use of PRA [ProbRA] would allow EPA to send the appropriate signal to the intellectual marketplace, thereby encouraging analysts to gather data and develop methodologies necessary for assessing uncertaintie*s” but also noticed: “*A clear institutional understanding of how to incorporate the results of probabilistic analyses into decision making is lacking*”. ProbRA is a form of data analysis making use of probabilities. There are four major data analytics disciplines^54^:

1. Descriptive analytics, e.g., for automated insights, large patterns, anomalous patterns, multivariate analysis
2. Diagnostic analytics, e.g., for value of information, reasoning, troubleshooting, tracing anomalies
3. Predictive analytics, e.g., for supervised or unsupervised learning, anomaly detection, time series, latent variables
4. Prescriptive analytics, e.g., for decision automation, cost-based decision-making, decision support, decision-making under uncertainty.

To some extent, ProbRA touches on all four aspects, but the central argument here is its use to predict risks. Toxicology would be well-served to address the value of probabilistic approaches in all of these.

ProbRA is a key element of the European flagship project ONTOX^55^ (Vinken et al., 2021) and the ASPIS cluster^56^ formed with two sister projects. ONTOX shall deliver a generic strategy to create innovative NAMs in order to predict systemic repeated dose toxicity effects that, upon combination with tailored exposure assessment, enable human risk assessment. The six specific adversities addressed are in the liver (steatosis and cholestasis), kidneys (tubular necrosis and crystallopathy) and developing brain (neural tube closure and cognitive function defects). A workshop on ProbRA jointly organized by CAAT through the transatlantic think tank for toxicology (t^4^)^57^ and ONTOX will further address this topic this summer. With a broad participation of regulators from both sides of the Atlantic in ASPIS, this promises to stimulate renewed discussion about ProbRA in regulatory sciences.

**Fig. 4:**
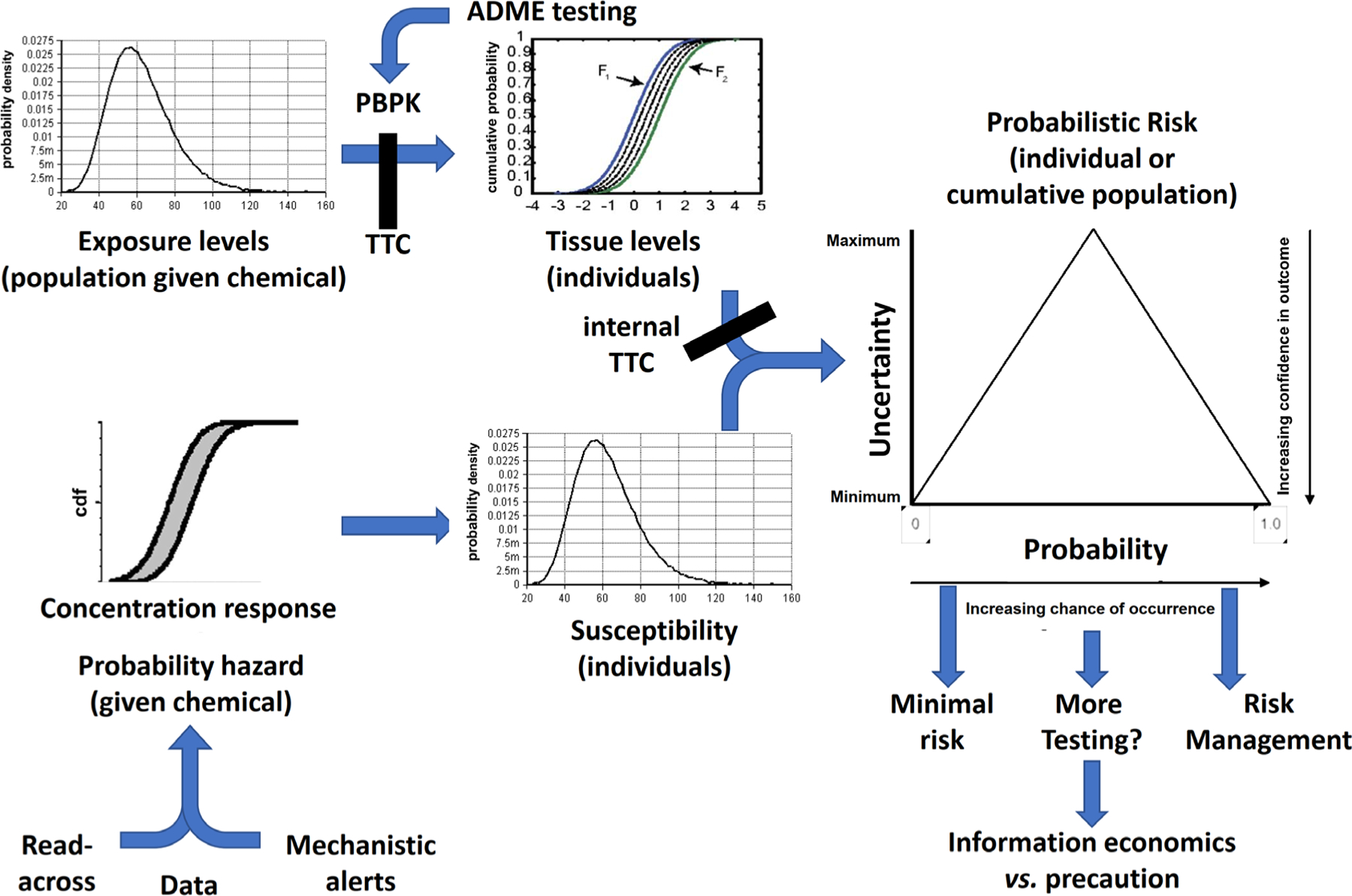
A vision for probabilistic risk assessment (ProbRA) of substances ProbRA is fueled by probability of exposure and probability of hazard and susceptibility. Exposure is first characterized by a population distribution (cumulative from the individuals’ exposure distributions). Where they do not exceed applicable thresholds of toxicological concern (TTC), the assessment might be abrogated on the ground of negligible exposure. Probabilistic physiology-based pharmacokinetic (or toxicokinetic, respectively) modeling (PBPK) translates these into resulting tissue concentrations. This can be refined by adsorption, metabolism, distribution & excretion (ADME) measurements or estimates. Internal TTC again might allow to abrogate the assessment in case of irrelevant tissue level concentrations. The second line of evidence is establishing the probability of hazard. This can be based on mechanistic data, mechanistic tests, and read-across to similar chemicals and any combination thereof. This probability is ideally combined with a distribution of susceptibility of different individuals. Together, tissue level concentrations and hazard probabilities give a probabilistic risk for an individual and cumulatively for the population. Low risk can lead to deprioritization depending on the use scenario, while high risk should lead to classification and risk management measures as appropriate. Intermediate probabilities of risk, i.e., high uncertainties, should be considered for additional testing, ideally considering the economics of possible information gain, or precautionary risk management.

Here, we would like to put forward a vision for ProbRA. Figure 4 shows the combination of the different probabilistic approaches above. Noteworthy, we see a key role for TTC to abrogate risk assessment where exposure and/or bioavailability (internal TTC) (Hartung and Leist, 2008; Partosch et al., 2015) is negligible. A.I. will play a key role for data extraction as well as for evidence integration. Here, especially Bayesian approaches lend themselves to the deduction of a probability of risk. Probability of hazard as the other starting point will be informed by data available on a given chemical including through (Q)SAR as well as data on similar chemicals through automated read-across. Here, we will build on the RASAR (Hartung, 2016; Luechtefeld et al., 2018a,b). An additional line of information on possible hazard will come from mechanistic alerts. The ontology approach of organizing such knowledge (Desprez et al., 2019) will be followed.

A key question for the future will be whether to employ a frequentist or Bayesian ProbRA? Based on the discussion of the Bayesian approach above, this seems to be most promising but might overwhelm risk assessment practitioners with its additional complexities. In areas like evidence integration by BN and similar, it might already sneak in as part of the data analysis procedures. A big limitation of machine learning models is causal inference. BN can sometimes handle that better. There are relationships between probabilistic inference and causal inference. If your training data has only been built within a certain environment, then machine learning models (and even probabilistic methods) can learn conditional probability relationships that are not valid – basically the same thing as saying correlation is not causation. It is worth mentioning that the problems A.I. has with learning probability distributions also can apply to animal testing, particularly methods like weight of evidence. Overall, there is great promise of Bayesian tools for risk assessment (Linkov et al., 2015).

## 16 Is ProbRA the keystone, the capstone, or the cornerstone of a new risk assessment?

**Fig. 5:**
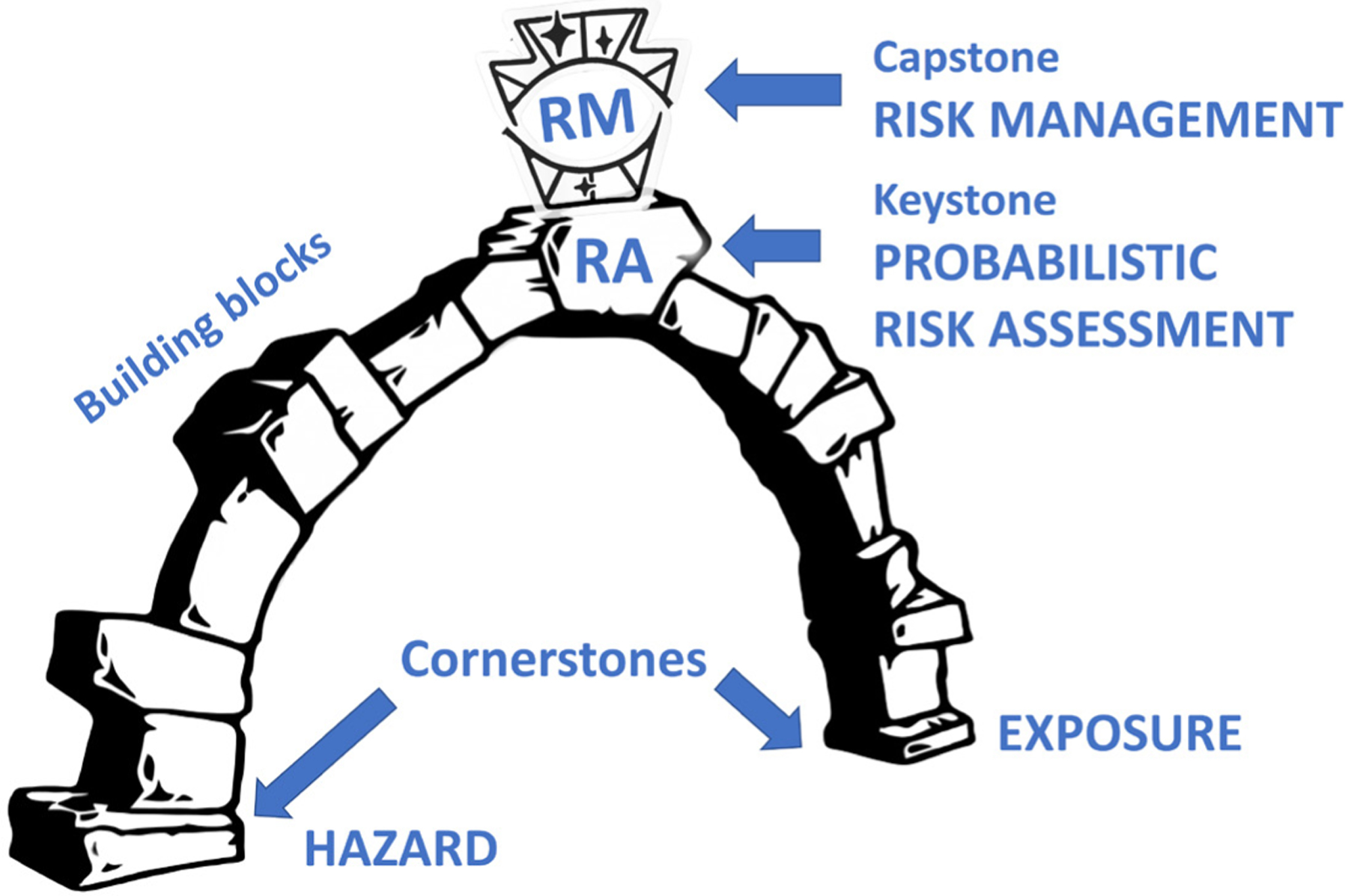
“Building” safety assessmentsby probabilistic risk assessment (ProbRA) Terminology from masonry was adapted to risk assessment to illustrate the integrating role of ProbRA. Graphic elements modified from: https://www.redbubble.com/shop/keystone+posters, https://pngset.com/download-free-png-yggdq

While well-defined in masonry^58^, these terms are sometimes used interchangeably in the figurative sense. It is worth thinking what the different terms mean relative to “building” the new toxicology (Fig. 5). The cornerstone, i.e., “*the first stone laid when constructing a masonry foundation. It is considered the most important stone in the building, as all other stones are laid in reference to this first, cornerstone*”, represents the hazards and exposures to protect against. The subsequent stones are the technologies and models, which allow to assess the two. As laid out above, ultimately, this leads to a probability of hazard and a probability of exposure for an individual by integration of the population. The two sides of the arch need to be combined by the keystone, i.e., “*the central stone placed at the top of an arch. The keystone is the apex of an arch, without it the arch would not stand. The keystone is placed last when constructing an arch, locking all the other stones into place*.” This is, in the authors’ view, the role of ProbRA, as the title of this article already gives away. Noteworthy, “*The word keystone is often used figuratively to mean the central idea of a philosophy, process, business proposition or principle upon which the entire philosophy, process, business proposition or principle stands*.”

What about the capstone then? “*A capstone is a finishing stone atop an exterior wall or roof or other exterior architectural feature. The capstone protects the masonry, causing water to flow in a certain way as to mitigate erosion*.” The best match would be the risk management implemented on the basis of probability of risk and policy decisions, i.e., what is best for society, the “polis”. As laid out above, it is tempting to call for this to be an *evidence-based risk management*.

Let’s close this reasoning about building ProbRA with a quote from the English author Walter Bagehot (1826–1877), “*Life is a school of probability*”. We are looking forward to making probability a greater part of the life of toxicologists.

## Figures and Tables

**Tab. 1:** Different risk types characterized by probability, possible damage, uncertainty, and public interest – iconic Greek mythology and (toxicological) examples

Greek mythology	Risk types	Examples	Toxicology examples
Sword of Damocles	low probability, large damage	Nuclear reactors, dams, chemical plants	Chemical spills
Cyclops	uncertain probability, large damage	Earthquake, flood, eruption, ABC weapons	Post-marketing drug failure
Pythia	uncertain probability, uncertain damage	Disintegration of polar ice sheets, GMO technology	Chemicals’ contribution to obesity, miscarriage, childhood asthma
Pandora’s box	uncertain probability, uncertain damage, unknown causal processes	Persistent organic pollutants, endocrine disruptors, ecosystem changes	dito; nanoparticle toxicity
Cassandra	high probability, high delayed damage	Global atmospheric warming, loss of biodiversity	Smoking, air pollution
Medusa	high public unrest, little scientific concern	Electromagnetic radiation (UMTS), food irradiation	Vaccine safety

Modified from Vlek (2010), derived from Klinke and Renn (2002); the authors added the column on toxicology.

**Tab. 2:** Key questions addressed in ProbRA and associated tools

Question	Tools
What can go wrong? Screen important initiators.	Master logic diagrams (MLD) or failure modes and effects analyses (FMEA); in toxicology, these would be relevant exposures or molecular initiating events (MIE) triggered within the adverse outcome pathway (AOP) framework
What are the adverse consequences?	Deterministic analyses that describe the phenomena that could occur along the path of the accident (here hazard) scenario. In toxicology, this can be understood as the exposure-to-hazard path, more recently defined as AOP with their key events (KE).
What is the probability of adverse consequences?	Boolean logic methods for model development (e.g., event tree analysis (ETA) or event sequence diagrams (ESD) analysis and deductive methods like fault tree analysis (FTA)) and by probabilistic or statistical methods for the quantification portion of the model analysis (deductive logic tools like fault trees or inductive logic tools like reliability block diagrams (RBD) and FMEA). The final result of a ProbRA is given in the form of a risk curve and the associated uncertainties. This is evidently least translated to toxicology.

**Tab. 3:** Non-comprehensive list of software packages for ProbRA and Monte Carlo simulations

Model	Developer/associated organization	Availability^a^
APROBA-Plus	WHO, RIVM^15^, Bokkers et al., 2017	Free
CARES (Cumulative and Aggregate Risk Evaluation System)	CARES NG Development Organization^16^	Free
ConsExpo	RIVM^17^	Free
DEEM-FCID/Calendex (Dietary Exposure Evaluation Model-Food Commodity Intake Database/Calendex)	US EPA^18^	Free
FDA-iRisk	Food and Drug Administration Center for Food Safety and Applied Nutrition (FDA/CFSAN), Joint Institute for Food Safety and Applied Nutrition (JIFSAN) and Risk Sciences International (RSI)^19^	Free
mc2d	Pouillot et al.^20^	Free
MCRA (Monte Carlo Risk Assessment)	RIVM, EFSA^21^	Free
PROcEED (Probabilistic Reverse dOsimetry Estimating Exposure Distribution)	US EPA^22^	Free
SHEDS (Stochastic Human Exposure and Dose Simulation)	US EPA^24^	Free
AuvTool, bootstrap simulation and two-dimensional Monte Carlo simulation	Foodrisk.org ^25^	Free
Agena Risk	Agena Ltd., Fenton and Neil, 2014	Commercial
Crystal Ball	Oracle^26^	Commercial
@Risk	Palisade^27^	Commercial

a List of available models adapted from US EPA^23^

**Tab. 4:** Some software tools available for physiologically-based kinetic modeling

Model	Developer/associated organization	Availability^a^
MEGen, a model equation generator (EG) linked to a parameter database	CEFIC LRI^29^	Free
RVIS – open access PBPK modelling platform	CEFIC LRI, George Loizou (HSE)^30^	Free
MERLIN-Expo, total exposure assessment chain	Ciffroy et al., 2016,^31^	Free
KNIME suite of tools	COSMOS Project (SEURAT-1)^32^, Sala Benito et al., 2017	Free
High-throughput toxicokinetics (httk)	US EPA, Wang, 2010	Free
PLETHEM (Population Lifecourse Exposure-To-Health-Effects Model Suite)	Scitovation^33^, Pendse et al., 2017	Free
Berkeley Madonna	Berkeley Madonna^34^	Commercial
MATLAB	MathWorks^35^	Commercial
Simcyp’s Population-based Simulator	Certara^36^	Commercial
Gastroplus/ADMET/PBPK PLUS	SimulationPlus^37^	Commercial
Computational Systems Biology Software Suite (PKSim), tools for the molecular level (MoBi), the organismal level (PK-Sim)	Open Systems Pharmacology^38^	Commercial

a List of available models adapted from Paini et al. (2017)

**Tab. 5:** Major workshops on physiology-based pharmacokinetic/toxicokinetic modeling (PBPK) for risk assessment

Workshop/reference	Brief summary
ECVAM: The use of biokinetics and *in vitro* methods in toxicological risk evaluation, 1995, Utrecht, The Netherlands (Blaauboer et al., 1996)	Recommendations to encourage and guide future work in the PBK model field. 1. Explore possibilities to integrate *in vitro* data into the models; 2. Models are built on a case-by-case basis; 3. Establish documentation to illustrate what is needed experimentally; 4. Availability of data required for constructing models; 5. Establish databases; 6. Refine the partition coefficient; 7. Penetration rate should be incorporated into PBK models (barriers information); 8. Biotransformation CYP P450 reactions and information should be included into the model; 9. Emphasis on species comparison (rodent versus human); 10. Target organs and metabolism; 11. *In vitro* systems should be a reliable representation of *in vivo*; 12. PBK models should include dynamics; 13. Validation of PBK models should be done with independent data set; 14. Evaluation of the different software; 15. Sensitivity analysis employed to identify potential source of errors
ECVAM: Physiologically based kinetic (PBK) modelling: Meeting the 3Rs agendas, 2005, Ispra, Italy (Bouvier d’Yvoire et al., 2007)	To better define the potential role of PBK modelling as a set of techniques capable of contributing to the 3Rs in the risk assessment process of chemicals; needs for technical improvements and applications; needs to increase understanding and acceptance by regulatory authorities of the capabilities and limitations of these models. The recommendations were categorized into i) quality of PBK modelling; ii) availability of reference data and models; and iii) development of testing strategy
EPA/NIEHS/CIIT/ INERIS: Uncertainty and variability in PBPK models, 2006, RTP, NC, USA (Barton et al., 2007)	Better statistical models and methods; better databases for physiological properties and their variation; explore a wide range of chemical space; training, documentation, and software.
The Mediterranean Agronomic Institute of Chania: The International Workshop on the Development of GMP for PBPK models, 2007, Crete, Greece (Loizou et al., 2008)	Clear descriptions of good practices for (1) model development, i.e., research and analysis activities, (2) model characterization, i.e., methods to describe how consistent the model is with biology and the strengths and limitations of available models and data such as sensitivity analyses, (3) model documentation, and (4) model evaluation, i.e., independent review that will assist risk assessors in their decisions of whether and how to use the models, and also for model developers to understand expectations of various model purposes, e.g., research versus application in risk assessment
EPAA & EURL ECVAM: Potential for further integration of toxicokinetic modelling into the prediction of *in vivo* dose-response curves without animal experiments, 2011, Joint Research Centre, Italy (Bessems et al., 2014)	The aim of the workshop was to critically appraise PBK modelling software platforms as well as a more detailed state-of-the-art overview of non-animal based PBK parameterization tools. Such as: 1) Identification of gaps in non-animal test methodology for the assessment of ADME. 2) Addressing user-friendly PBK software tools and free-to-use web applications. 3) Understanding the requirements for wider and increased take up and use of PBK modelling by regulators, risk assessors and toxicologists in general. 4) Tackling the aspect of obtaining *in vivo* human toxicokinetic reference data via micro-dosing following the increased interest by the research community, regulators, and politicians
US FDA: Application of Physiologically-based pharmacokinetic (PBPK) modelling to support dose selection, 2014, Silver Spring, MD, USA (Wagner et al., 2015)	Workshop to (i) assess the current state of knowledge in the application of PBK in regulatory decision-making, and (ii) share and discuss best practices in the use of PBK modelling to inform dose selection in specific patient populations
EURL ECVAM: Physiologically-based kinetic modelling in risk assessment – Reaching a whole new level in regulatory decision-making, 2016, Joint Research Centre, Italy (Paini et al., 2017)	Strategies to enable prediction of systemic toxicity by applying new approach methodologies (NAM) using PBK modelling to integrate *in vitro* and *in silico* methods for ADME in humans for predicting whole-body TK behavior, for environmental chemicals, drugs, nano-materials, and mixtures. (i) identify current challenges in the application of PBK modelling to support regulatory decision-making; (ii) discuss challenges in constructing models with no *in vivo* kinetic data and opportunities for estimating parameter values using *in vitro* and *in silico* methods; (iii) present the challenges in assessing model credibility relying on non-animal data and address strengths, uncertainties and limitations in such an approach; (iv) establish a good kinetic modelling practice workflow to serve as the foundation for guidance on the generation and use of *in vitro* and *in silico* data to construct PBK models designed to support regulatory decision making. Recommendations on parameterization and evaluation of PBK models: (i) develop a decision tree for model construction; (ii) set up a task force for independent model peer review; (iii) establish a scoring system for model evaluation; (iv) attract additional funding to develop accessible modelling software; (v) improve and facilitate communication between scientists (model developers, data provider) and risk assessors/regulators; and (vi) organize specific training for end users. Critical need for developing a guidance document on building, characterizing, reporting, and documenting PBK models using non-animal data; incorporating PBK models in integrated strategy approaches and integrating them with *in vitro* toxicity testing and adverse outcome pathways.

**Tab. 6:** Examples of ProbRA in toxicology

Topic of ProbRA	Reference
Agrochemicals in the environment	Solomon et al., 2000
Pesticide atrazine in the environment	Verdonck et al., 2002
Environmentally occurring pharmaceuticals	Sanderson, 2003
Linear alkylbenzene sulfonate (LAS) in sewage sludge	Schowanek et al., 2007
Chemical constituents in mainstream smoke of cigarettes	Xie et al., 2012
Flame retardant PBDE in fish	Pardo et al., 2014
Insecticides (malathion and permethrin)	Schleier et al., 2015
Nanosilica in food	Jacobs et al., 2015
Reproductive and developmental toxicants in consumer products	Durand et al., 2015
Perfluorooctane sulfonate (PFOS)	Chou and Lin, 2020

**Tab. 7:** Advantages and challenges for ProbRA in human health risk assessment

Advantages of ProbRA	Challenges of ProbRA
Improves **transparency and credibility** by explicit consideration and treatment of all types of uncertainties; clearly structured; integrative and quantitative; allows ranking of issues and results; more information can be obtained by separating variability from uncertainty	Problem of **model incompleteness**; relatively time-consuming in performing and interpreting – this “*might be a fertile ground for endless debate between utility and regulator*” (Kafka, 1998); regulatory delays due to the necessity of analyzing numerous scenarios using various models
**Cost effective** by assuring that resources are focused on essential safety issues, focuses data collection	More **complex and time-consuming** analysis and decision-making process because more information and insights must be collected, processed and considered for decisions; **requires more data** than conventional approaches because distributions of values rather than single values are used
**More realistic** compared to the current deterministic RA: avoids worst-case assumptions, realistic exposure assessments; overall picture of risks in the population and not just of extreme cases; a probabilistic reference dose could help reduce the potentially inaccurate implication of zero risk below the reference dose.	The **incompleteness** of the model is much **more “apparent”**
**Improves decision support** enabling risk managers to evaluate the full range of variability and uncertainty instead of just using point estimates of exposure, effects, and eventually risk.	More complex structure, the assumptions, methods and results are more difficult to understand and require some **mathematical education**; lack of understanding of the value of ProbRA for decision-making; personnel must be very well-informed scientifically and technologically to produce consistent application of standards; requires a different skill set than used in current evaluations, but limited resources (staff, time, training or methods) are available
Includes a **systematic sensitivity analysis** of the uncertainties in the input parameters, which identifies the main sources of uncertainty. Sensitivity analysis is the study of how uncertainty in the output of a model (numerical or otherwise) can be apportioned to different sources of uncertainty in the model input (Saltelli et al., 2008).	Where **extremely rare events** must be considered, there are problems with the statistical significance of probabilistic data
Application of an **optimization process** (Apostolakis, 1990)	**Validation challenge**; what to compare against? **Good practices lacking**
**More effective risk management;** enhances safety and helps manage operability; estimating the success of intervention measures is improved	**Complicates decision-making** where a more comprehensive characterization of the uncertainties leads to a decrease in clarity regarding how to estimate risk for the scenario under consideration
**More transparent risk communication:** results and decisions can be communicated on a clearly defined basis	**Communicating** ProbRA and the impact on the decision/policy options is **complex**; results are characterized as prognostic estimations of what can or cannot happen in future makes understanding difficult and poses a still **unresolved issue for many legal environments**
**Works with limited data:** Even if the amount of available adequate probabilistic data is relatively small, the absolute accuracy of the data is not an issue if probabilistic approaches are used as comparative tools, allowing one to make decisions between different design or operation alternatives	**Minimum data requirements** currently are a topic of debate; any **quantitative risk estimate** only makes sense when the employed data are statistically significant in a sense (i.e., sufficient observations available) and if they originate from similar events and have been analyzed with respect to a common criterion
**Information economy:** Enables estimating formally the value of gathering more information; better prioritize information needs by investing in areas that yield the greatest information value	Difficulty to **quantify and weigh risks and benefits**
	**Various communities** have unique sets of perspectives, historical practices, terminologies, resources, and propensities, governed by overlapping set(s) of problems and decision-making goals, regulatory requirements, and legislative mandates being addressed, directly or indirectly, by these interrelated communities.

## Box 1: The U.S. Environmental Protection Agency Conditions for Acceptance of ProbRA8

1. The purpose and scope of the assessment should be clearly articulated in a “problem formulation” section that includes a full discussion of any highly exposed or highly susceptible subpopulations evaluated (e.g., children, the elderly). The questions the assessment attempts to answer are to be discussed and the assessment endpoints are to be well defined.
2. The methods used for the analysis (including all models used, all data upon which the assessment is based, and all assumptions that have a significant impact upon the results) are to be documented and easily located in the report. This documentation is to include a discussion of the degree to which the data used are representative of the population under study. Also, this documentation is to include the names of the models and software used to generate the analysis. Sufficient information is to be provided to allow the results of the analysis to be independently reproduced.
3. The results of sensitivity analyses are to be presented and discussed in the report. Probabilistic techniques should be applied to the compounds, pathways, and factors of importance to the assessment, as determined by sensitivity analyses or other basic requirements of the assessment.
4. The presence or absence of moderate to strong correlations or dependencies between the input variables is to be discussed and accounted for in the analysis, along with the effects these have on the output distribution.
5. Information for each input and output distribution is to be provided in the report. This includes tabular and graphical representations of the distributions (e.g., probability density function and cumulative distribution function plots) that indicate the location of any point estimates of interest (e.g., mean, median, 95^th^ percentile). The selection of distributions is to be explained and justified. For both the input and output distributions, variability and uncertainty are to be differentiated where possible.
6. The numerical stability of the central tendency and the higher end (i.e., tail) of the output distributions are to be presented and discussed.
7. Calculations of exposures and risks using deterministic (e.g., point estimate) methods are to be reported if possible. Providing these values will allow comparisons between the probabilistic analysis and past or screening level risk assessments. Further, deterministic estimates may be used to answer scenario-specific questions and to facilitate risk communication. When comparisons are made, it is important to explain the similarities and differences in the underlying data, assumptions, and models.
8. Since fixed exposure assumptions (e.g., exposure duration, body weight) are sometimes embedded in the toxicity metrics (e.g., reference doses, reference concentrations, unit cancer risk factors), the exposure estimates from the probabilistic output distribution are to be aligned with the toxicity metric.

## Footnotes

1 Supposedly first used by Daniel Defoe, in *The Political History of the Devil*, 1726: “*Things as certain as death and taxes, can be more firmly believed*.”

2 Thanks to Kimbal Atwood and his blogs Science-based Medicine bringing them to our attention: https://sciencebasedmedicine.org/author/kimball-atwood/

3
https://www.psychologicalscience.org/observer/bayes-for-beginners-probability-and-likelihood

4 William K. Reilly: Aiming Before We Shoot: The Quiet Revolution in Environmental Policy; Address to the National Press Club on September 26, 1990

5 Taleb (2007) “*What is surprising is not the magnitude of our forecast errors, but our absence of awareness of it”; “True, our knowledge does grow, but it is threatened by greater increases in confidence, which makes our increase in knowledge at the same time an increase in confusion, ignorance, and conceit*”.

6
https://www.ebtox.org

7
https://copswiki.org/w/pub/Common/M1922/pra%20-%20Probabilistic%20Risk%20Assessment%20-%20What%20is%20it%20and%20why%20is%20it%20work%20performing.pdf

8
https://www.epa.gov/sites/default/files/2014-11/documents/probpol.pdf

9
https://towardsdatascience.com/a-gentle-introduction-to-maximum-likelihood-estimation-9fbff27ea12f

10
https://www.aptech.com/blog/beginners-guide-to-maximum-likelihood-estimation-in-gauss/

11 This cited point does not really hold any more given the continuous increase in computing power.

12
https://www.bayesserver.com/docs/introduction/bayesian-networks

13
https://www.bayesfusion.com/bayesian-networks/

14
https://www.epa.gov/sites/default/files/2014-12/documents/raf-pra-white-paper-final.pdf

15
https://www.rivm.nl/en/aproba-plus

16
https://caresng.org

17
https://www.rivm.nl/en/consexpo

18
https://www.epa.gov/pesticide-science-and-assessing-pesticide-risks/deem-fcidcalendex-software-installer

19
https://irisk.foodrisk.org

20
https://cran.r-project.org/web/packages/mc2d/vignettes/docmcEnglish.pdf

21
https://mcra.rivm.nl/Select

22
https://www.epa.gov/chemical-research/probabilistic-reverse-dosimetry-estimating-exposure-distribution-proceed

23
https://www.epa.gov/expobox/exposure-assessment-tools-tiers-and-types-deterministic-and-probabilistic-assessments

24
https://www.epa.gov/chemical-research/stochastic-human-exposure-and-dose-simulation-sheds-estimate-human-exposure

25
https://www.foodrisk.org/resources/display/57

26
https://www.oracle.com/applications/crystalball/

27
https://www.palisade.com/risk/

28
https://www.epa.gov/expobox/exposure-assessment-tools-tiers-and-types-deterministic-and-probabilistic-assessments

29
http://cefic-lri.org/toolbox/pbpkmegen/

30
http://cefic-lri.org/projects/aimt7-rvis-open-access-pbpk-modelling-platform/

31
https://merlin-expo.eu/

32
http://www.cosmostox.eu/what/webtutorials/

33
http://www.scitovation.com/plethem.html

34
https://berkeley-madonna.myshopify.com

35
https://www.mathworks.com/products/matlab.html

36
https://www.certara.com/software/pbpk-modeling-and-simulation/physiologically-based-pharmacokinetic-modeling-and-simulation/

37 http://www.simulations-plus.com/software/gastroplus/; https://www.simulations-plus.com/software/admetpredictor; https://www.simulations-plus.com/software/pkplus/

38 https://www.open-systems-pharmacology.org; https://github.com/Open-Systems-Pharmacology/Suite

39
https://www.efsa.europa.eu/en/efsajournal/pub/2839

40
http://www.bfr.bund.de/en/probabilistic_exposure_assessment-10420.html

41
https://www.epa.gov/chemical-research/rapid-chemical-exposure-and-dose-research

42
https://www.ul.com/services/predictive-toxicology-solutions

43
https://www.legislation.gov.au/Details/C2019A00012

44
https://www.eu-toxrisk.eu

45
https://www.epa.gov/chemical-research/toxicity-forecasting

46
https://www.epa.gov/sites/default/files/2018-09/documents/genra_help_310818.pdf

47
https://aopwiki.org/wiki/index.php/Aop:40

48
https://siliconangle.com/blog/2018/04/20/digital-twins-personalized-medicine-promising-caveats/

49
https://www.philips.com/a-w/about/news/archive/blogs/innovation-matters/20181211-the-digital-patient-will-we-one-day-have-our-own-health-avatars.html

50
http://www.digital-patient.net

51
https://www.vph-institute.org/upload/discipulus-digital-patient-research-roadmap_5270f44c03856.pdf

52
https://towardsdatascience.com/making-your-neural-network-say-i-dont-know-bayesian-nns-using-pyro-and-pytorch-b1c24e6ab8cd

53
http://www.bfr.bund.de/en/probabilistic_exposure_assessment-10420.html

54 Modified from: https://www.bayesserver.com/docs/introduction/bayesian-networks

55
https://ontox-project.eu

56
https://www.aspis-cluster.com

57
https://caat.jhsph.edu/about/t4.html

58
https://grammarist.com/usage/capstone-keystone-or-cornerstone/

## Glossary and abbreviations

Extracted from Last (2001), EPA (2014, 1999), EFSA (2018), Ferrario et al. (2014) and^59^

ADME: Generally used as the abbreviation for absorption, distribution, metabolism, excretion.
Adverse outcome pathway (AOP): An AOP is a sequence of events from the exposure of an individual or population to a chemical substance through a final adverse (toxic) effect at the individual level (for human health) or population level (for ecotoxicological endpoints). The key events in an AOP should be definable and make sense from a physiological and biochemical perspective. AOPs incorporate the toxicity pathway and mode of action for an adverse effect. AOPs may be related to other mechanisms and pathways as well as to detoxification routes.
Applicability domain: The physicochemical, structural, or biological space and information that was used to develop a (Q)SAR model and for which that model gives predictions with a given level of reliability.
Bias: A systematic error or deviation in results or inferences from the truth.
Biokinetics (in toxicology): Science of the movements involved in the distribution of substances.
Biomarker: Indicator signaling an event or condition in a biological system or sample and giving a measure of exposure, effect, or susceptibility.
Data analysis procedure (DAP): DAP refers to a procedure incorporating both a data interpretation procedure (DIP) and a prediction model (PM).
Deterministic: A methodology relying on point (i.e., exact) values as inputs to estimate risk; this obviates quantitative estimates of uncertainty and variability. Results also are presented as point values. Uncertainty and variability may be discussed qualitatively or semi-quantitatively by multiple deterministic risk estimates.
Frequentist (or frequency) probability: A view of probability that concerns itself with the frequency with which an event occurs given a long sequence of identical and independent trials.
Hazard: 1) A biological, chemical, or physical agent with the potential to cause an adverse health effect. 2) The inherent characteristic of a material, condition, or activity that has the potential to cause adverse effects to people, property, or the environment.
Hazard identification: The risk assessment process of determining whether exposure to a stressor can cause an increase in the incidence or severity of a particular adverse effect, and whether an adverse effect is likely to occur.
Integrated testing strategy (ITS): In the context of safety assessment, an integrated testing strategy is a methodology which integrates information for toxicological evaluation from more than one source, thus facilitating decision-making. This should be achieved whilst taking into consideration the principles of the Three Rs (reduction, refinement, and replacement).
Likelihood ratio: The likelihood that a given test result would be expected in a patient with the target disorder compared to the likelihood that the same result would be expected in a patient without that disorder. for a positive test result = LR+ = sensitivity/(1-specificity) for a negative test result = LR− = (1-sensitivity)/specificity
Model: A mathematical representation of a natural system intended to mimic the behavior of the real system, allowing description of empirical data and predictions about untested states of the system.
Modeling: Development of a mathematical or physical representation of a system or theory that accounts for all or some of its known properties. Models often are used to test the effect of changes of components on the overall performance of the system.
Monte Carlo analysis or simulation: A repeated random sampling from the distribution of values for each of the parameters in a generic exposure or risk equation to derive an estimate of the distribution of exposures or risks in the population.
NAM: New approach methodologies
One-dimensional Monte Carlo analysis: A method for making probability calculations by random sampling from one set of distributions, all representing uncertainty about non-variable quantities or categorical questions. A numerical method of simulating a distribution for an endpoint of concern as a function of probability distributions that characterize variability or uncertainty. Distributions used to characterize variability are distinguished from distributions used to characterize uncertainty.
Parameter: A quantity used to calibrate or specify a model, such as “parameters” of a probability model (e.g., mean and standard deviation for a normal distribution). Parameter values often are selected by fitting a model to a calibration data set.
Physiologically-based pharmacokinetic models (PBPK): A computer model that describes what happens to a chemical in the body. This model describes how the chemical gets into the body, where it goes in the body, how it is changed by the body, and how it leaves the body.
Probability: Defined depending on philosophical perspective: (1) the frequency with which sampled values arise within a specified range or for a specified category; (2) quantification of judgement regarding the likelihood of a particular range or category. A frequentist approach considers the frequency with which samples are obtained within a specified range or for a specified category (e.g., the probability that an average individual with a particular mean dose will develop an illness).
Probability density function: In probability theory, a probability density function (pdf) of a continuous random variable is a function, often denoted as f(x), that describes the relative likelihood for this random variable to take on a given value.
Probabilistic modeling: A technique that utilizes the entire range of input data to develop a probability distribution of exposure to risk rather than a single point value. The input data can be measured values and/or estimated distributions. Values for these input parameters are sampled thousands of times through a modeling or simulation process in order to develop a distribution of likely exposure or risk. Probabilistic models can be used to evaluate the impact of variability and uncertainty in the various input parameters, such as environmental exposure levels, fate, and transport processes.
Probabilistic risk analysis (ProbRA): A risk assessment that uses probabilistic methods (e.g., Monte Carlo analysis) to derive a distribution of risk based on multiple sets of values sampled for random variables. Calculation and expression of health risks using multiple risk descriptors to provide the likelihood of various risk levels. Probabilistic risk results approximate a full range of possible outcomes and the likelihood of each, which often is presented as a frequency distribution graph, thus allowing uncertainty or variability to be expressed quantitatively.
QIVIVE: Quantitative in vitro to in vivo extrapolation
Quantitative structure-activity relationship (QSAR): A QSAR is a theoretical model for making predictions of physicochemical properties, environmental fate parameters, or biological effects (including toxic effects in environmental and mammalian species). QSARs relate quantitative measures of chemical structure to continuous or categorical variables describing the property to be predicted.
Risk: A measure of the probability that damage to life, health, property, and/or the environment will occur as a result of a given hazard. 1. Risk includes consideration of exposure to the possibility of an adverse outcome, the frequency with which one or more types of adverse outcomes may occur, and the severity or consequences of the adverse outcomes if such occur. 2. The potential for realization of unwanted, adverse consequences to human life, health, property, or the environment. 3. The probability of adverse effects resulting from exposure to an environmental agent or mixture of agents. 4. The combined answers to: What can go wrong? How likely is it? What are the consequences?
Risk analysis: A process for identifying, characterizing, controlling, and communicating risks in situations where an organism, system, subpopulation, or population could be exposed to a hazard. Risk analysis is a process that includes risk assessment, risk management and risk communication.
Risk assessment: Qualitative and quantitative evaluation of the risk posed to human health and/or the environment by the actual or potential presence and/or use of specific pollutants 1. A process intended to calculate or estimate the risk to a given target organism, system, subpopulation, or population, including the identification of attendant uncertainties following exposure to a particular agent, taking into account the inherent characteristics of the agent of concern, as well as the characteristics of the specific target system. 2. The evaluation of scientific information on the hazardous properties of environmental agents (hazard characterization), the dose-response relationship (dose-response assessment), and the extent of human exposure to those agents (exposure assessment). The product of the risk assessment is a statement regarding the probability that populations or individuals so exposed will be harmed and to what degree (risk characterization). 3. Qualitative and quantitative evaluation of the risk posed to human health or the environment by the actual or potential presence or use of specific pollutants.
Risk management: A decision-making process that takes into account environmental laws, regulations, and political, social, economic, engineering and scientific information, including a risk assessment, to weigh policy alternatives associated with a hazard.
Sensitivity analysis: The process of changing one variable while leaving the others constant to determine its effect on the output. This procedure fixes each uncertain quantity at its credible lower and upper bounds (holding all others at their nominal values, such as medians) and computes the results of each combination of values. The results help to identify the variables that have the greatest effect on exposure estimates and help focus further information-gathering efforts.
Two-dimensional Monte Carlo analysis: A method for making probability calculations by random sampling from two sets of distributions, one set describing the variability of variable quantities, and the second set representing uncertainty, including uncertainty about the parameters of the distributions describing variability. An advanced numerical modeling technique that uses two stages of random sampling, also called nested loops, to distinguish between variability and uncertainty in exposure and toxicity variables. The first stage, often called the inner loop, involves a complete 1-D MCA simulation of variability in risk. In the second stage, often called the outer loop, parameters of the probability distributions are redefined to reflect uncertainty. These loops are repeated many times resulting in multiple risk distributions, from which confidence intervals are calculated to represent uncertainty in the population distribution of risk.
Uncertainty: Uncertainty occurs because of a lack of knowledge. It is not the same as variability. For example, a risk assessor may be very certain that different people drink different amounts of water but may be uncertain about how much variability there is in water intakes within the population. Uncertainty often can be reduced by collecting more and better data, whereas variability is an inherent property of the population being evaluated. Variability can be better characterized with more data, but it cannot be reduced or eliminated. Efforts to clearly distinguish between variability and uncertainty are important for both risk assessment and risk characterization, although they both may be incorporated into an assessment.
Uncertainty analysis: A detailed examination of the systematic and random errors of a measurement or estimate; an analytical process to provide information regarding uncertainty.
Value of information: An analysis that involves estimating the value that new information can have to a risk manager before the information is actually obtained. It is a measure of the importance of uncertainty in terms of the expected improvement in a risk management decision that might come from better information.
Variability: Refers to true heterogeneity or diversity, as exemplified in natural variation. For example, among a population that drinks water from the same source and with the same contaminant concentration, the risks from consuming the water may vary. This may result from differences in exposure (e.g., different people drinking different amounts of water and having different body weights, exposure frequencies and exposure durations), as well as differences in response (e.g., genetic differences in resistance to a chemical dose). Those inherent differences are referred to as variability. Differences among individuals in a population are referred to as inter-individual variability, and differences for one individual over time are referred to as intra-individual variability.

## References

[R1] Al-Chalabi A and Hardiman O (2013). The epidemiology of ALS: A conspiracy of genes, environment and time. Nat Rev Neurol 9, 617–628. doi:10.1038/nrneurol.2013.203 24126629

[R2] Aldenberg T and Jaworska JS (2010). Multiple test in silico weight-of-evidence for toxicological endpoints. Issues Toxicol 7, 558–583.

[R3] Apostolakis G (1990). The concept of probability in safety assessment of technological systems. Science 50, 1359–1366. doi: 10.1126/science.2255906 2255906

[R4] Aughenbaugh JM and Paredis CJJ (2006). The value of using imprecise probabilities in engineering design. J Mech Des 128, 969–979. doi:10.1115/1.2204976

[R5] Barton HA, Chiu WA, Setzer RW (2007). Characterizing uncertainty and variability in physiologically-based pharmacokinetic (PBPK) models: State of the science and needs for research and implementation. Toxicol Sci 99, 395–402. doi:10.1093/toxsci/kfm100 17483121

[R6] Basketter DA, Clewell H, Kimber I (2012). A roadmap for the development of alternative (non-animal) methods for systemic toxicity testing. ALTEX 29, 3–89. doi:10.14573/altex.2012.1.003 22307314

[R7] Bessems JG, Loizou G, Krishnan K (2014). PBTK modelling platforms and parameter estimation tools to enable animal-free risk assessment: Recommendations from a joint EPAA-EURL ECVAM ADME workshop. Regul Toxicol Pharmacol 68, 119–139. doi:10.1016/j.yrtph.2013.11.008 24287156

[R8] Blaauboer B, Bayliss MK, Castell J (1996). The use of biokinetics and in vitro methods in toxicological risk evaluation. The report and recommendations of ECVAM Workshop 15. Altern Lab Anim 24, 473–497. doi:10.1177/026119299602400408

[R9] Bogen KT and Spear RC (1987). Integrating uncertainty and interindividual variability in environmental risk assessment. Risk Anal 7, 427–436. doi:10.1111/j.1539-6924.1987.tb00480.x 3444930

[R10] Bogen KT and Hall LC (1989). Pharmacokinetics for regulatory risk analysis: The case of 1,1,1-trichloroethane (methyl chloroform). Regul Toxicol Pharmacol 10, 26–50. doi:10.1016/0273-2300(89)90011-1 2672126

[R11] Bogen KT, Cullen AC, Frey HC (2009). Probabilistic exposure analysis for chemical risk characterization. Toxicol Sci 109, 4–17. doi:10.1093/toxsci/kfp036 19223660 PMC3692252

[R12] Bokkers B, Mengelers MJ, Bakker MI (2017). APROBA-Plus: A probabilistic tool to evaluate and express uncertainty in hazard characterization and exposure assessment of substances. Food Chem Toxicol 110, 408–417. doi:10.1016/j.fct.2017.10.038 29074418 PMC5793872

[R13] Bottini AA and Hartung T (2009). Food for thought … on economics of animal testing. ALTEX 26, 3–16. doi:10.14573/altex.2009.1.3 19326029

[R14] Bouvier d’Yvoire M, Prieto P, Blaauboer BJ (2007). Physiologically-based kinetic modelling (PBK modelling): Meeting the 3Rs agenda. The report and recommendations of ECVAM Workshop 63. Altern Lab Anim 35, 661–671. doi: 10.1177/026119290703500606 18186671

[R15] Brown SA (2016). Principles for developing patient avatars in precision and systems medicine. Front Genet 6, 365. doi:10.3389/fgene.2015.00365 26779255 PMC4705226

[R16] Browne P, Judson RS, Casey WM (2015). Screening chemicals for estrogen receptor bioactivity using a computational model. Environ Sci Technol 49, 8804–8814. doi:10.1021/acs.est.5b02641 26066997

[R17] Brozek JL, Canelo-Aybar C, Akl EA (2021). GRADE Guidelines 30: The GRADE approach to assessing the certainty of modeled evidence – An overview in the context of health decision-making. J Clin Epidemiol 129, 138–150. doi:10.1016/j.jclinepi.2020.09.018 32980429 PMC8514123

[R18] Bruynseels K, Santoni De Sio F and van den Hoven J (2018). Digital twins in health care: Ethical implications of an emerging engineering paradigm. Front Genet 9, 31. doi:10.3389/fgene.2018.00031 29487613 PMC5816748

[R19] Chesnut M, Yamada T, Adams T (2018). Regulatory acceptance of read-across: Report from an international satellite meeting at the 56^th^ Annual Meeting of the Society of Toxicology. ALTEX 35, 413–419. doi:10.14573/altex.1805081 30008009

[R20] Chiu WA and Slob W (2015). A unified probabilistic framework for dose-response assessment of human health effects. Environ Health Perspect 123, 1241–1254. doi:10.1289/ehp.1409385 26006063 PMC4671238

[R21] Chou W-C and Lin Z (2020). Probabilistic human health risk assessment of perfluorooctane sulfonate (PFOS) by integrating in vitro, in vivo toxicity, and human epidemiological studies using a Bayesian-based dose-response assessment coupled with physiologically based pharmacokinetic (PBPK) modeling approach. Environ Int 137, 105581. doi:10.1016/j.envint.2020.105581 32087483

[R22] Ciffroy P, Alfonso B, Altenpohl A (2016). Modelling the exposure to chemicals for risk assessment: a comprehensive library of multimedia and PBPK models for integration, prediction, uncertainty and sensitivity analysis – The MERLIN-Expo tool. Sci Total Environ 568, 770–784. doi:10.1016/j.scitotenv.2016.03.191 27169730

[R23] Cristea IA and Ioannidis JPA (2018). P values in display items are ubiquitous and almost invariably significant: A survey of top science journals. PLoS One 13, e0197440. doi:10.1371/journal.pone.0197440 29763472 PMC5953482

[R24] Cullen AC and Frey HC (1999). Probabilistic Techniques in Exposure Assessment. A Handbook for Dealing with Variability and Uncertainty in Models and Inputs. New York, USA: Plenum.

[R25] Davison AC and Hinkley DV (1997). Bootstrap Methods and Their Application. Cambridge University Press.

[R26] De Vries RBM, Angrish M, Browne P (2021). Applying evidence-based methods to the development and use of adverse outcome pathways. ALTEX 38, 336–347. doi:10.14573/altex.2101211 33837437 PMC9394185

[R27] Desprez B, Birk B, Blaauboer B (2019). A mode-of-action ontology model for safety evaluation of chemicals: Outcome of a series of workshops on repeated dose toxicity. Toxicol In Vitro 59, 44–50. doi:10.1016/j.tiv.2019.04.005 30954655

[R28] Dirven H, Vist GE, Bandhakavi S (2021). Performance of preclinical models in predicting drug-induced liver injury in humans: A systematic review. Sci Rep 11, 6403. doi:10.1038/s41598-021-85708-2 33737635 PMC7973584

[R29] Dupuy J-P (1982). Ordres et Désordres: Enquête Sur un Nouveau Paradigme. Paris, France: Seuil.

[R30] Durand E, Leroux C, Perouel G, Beausoleil C (2015). Probabilistic risk assessment of consumer exposure to reproductive and developmental toxicants. J Pharmacol Clin Toxicol 3, 1049.

[R31] Efron B and Tibshirani R (1993). An Introduction to the Bootstrap. Boca Raton, FL, USA: Chapman & Hall/CRC.

[R32] EFSA (European Food Safety Authority) and EBTC (Evidence-Based Toxicology Collaboration) (2018). EFSA Scientific Colloquium 23: Evidence integration in risk assessment: The science of combining apples and oranges. EFSA Supporting Publication 16, EN-1396. doi:10.2903/sp.efsa.2018.EN-1396

[R33] EFSA Scientific Committee, Benford D, Halldorsson T (2018). Guidance on uncertainty analysis in scientific assessments. EFSA J 16, 5123. doi:10.2903/j.efsa.2018.5123 PMC7009727 32625671

[R34] EPA, U.S. (2014). Probabilistic Risk Assessment to Inform Decision Making: Frequently Asked Questions. https://www.epa.gov/sites/default/files/2014-11/documents/raf-pra-faq-final.pdf

[R35] Farhat N, Tsaioun K, Saunders-Hastings P (2022). Systematic review in evidence-based risk assessment. ALTEX, online ahead of print. doi:10.14573/altex.2004111 34585732

[R36] Fenton NE and Neil M (2014). Decision support software for probabilistic risk assessment using Bayesian networks. IEEE Software 31, 21–26. doi:10.1109/MS.2014.32

[R37] Ferrario D, Brustio R and Hartung T (2014). Glossary of reference terms for alternative test methods and their validation. ALTEX 31, 319–335. doi:10.14573/altex.1403311 24819604

[R38] Fisher JW, Gearhart JM and Lin Z (2020). Physiologically Based Pharmacokinetic (PBPK) Modeling – Methods and Applications in Toxicology and Risk Assessment. Academic Press, Elsevier. doi:10.1016/C2018-0-03297-1

[R39] Gilmour N, Kern PS, Alépée N (2020). Development of a next generation risk assessment framework for the evaluation of skin sensitisation of cosmetic ingredients. Regul Toxicol Pharma col 116, 104721. doi:10.1016/j.yrtph.2020.104721 32645429

[R40] Goodman SN (1999a). Toward evidence-based medical statistics. 1: The p value fallacy. Ann Intern Med 130, 995–1004. doi:10.7326/0003-4819-130-12-199906150-00008 10383371

[R41] Goodman SN (1999b). Toward evidence-based medical statistics. 2: The Bayes factor. Ann Intern Med 130, 1005–1013. doi:10.7326/0003-4819-130-12-199906150-00019 10383350

[R42] Hartung T, and Leist M (2008). Food for thought … on the evolution of toxicology and phasing out of animal testing. ALTEX 25, 91–96. doi:10.14573/altex.2008.2.91 18551232

[R43] Hartung T and Hoffmann S (2009). Food for thought on … in silico methods in toxicology. ALTEX 26, 155–166. doi:10.14573/altex.2009.3.155 19907903

[R44] Hartung T and McBride M (2011). Food for thought … on mapping the human toxome. ALTEX 28, 83–93. doi:10.14573/altex.2011.2.083 21625825

[R45] Hartung T (2013). Look back in anger – What clinical studies tell us about preclinical work. ALTEX 30, 275–291. doi:10.14573/altex.2013.3.275 23861075 PMC3790571

[R46] Hartung T, Luechtefeld T, Maertens A (2013). Integrated testing strategies for safety assessments. ALTEX 30, 3–18. doi:10.14573/altex.2013.1.003 23338803 PMC3800026

[R47] Hartung T (2016). Making big sense from big data in toxicology by read-across. ALTEX 33, 83–93. doi:10.14573/altex.1603091 27032088

[R48] Hartung T (2017a). A comprehensive overview of the current status and application of predictive ADMET: Introduction and Overview. In Chackalamannil S, Rotella D and Ward SE (eds.), Comprehensive Medicinal Chemistry III – Experimental ADME and Toxicology (Chapter 4–08, 150–155). doi:10.1016/B978-0-12-409547-2.12378-9

[R49] Hartung T (2017b). Thresholds of toxicological concern – Setting a threshold for testing where there is little concern. ALTEX 34, 331–351. doi:10.14573/altex.1707011 28735337

[R50] Hartung T (2017c). Utility of the adverse outcome pathway concept in drug development. Exp Opin Drug Metabol Toxicol 13, 1–3. doi:10.1080/17425255.2017.1246535 27718748

[R51] Hartung T (2018a). Perspectives on in vitro to in vivo extrapolations. J Appl In Vitro Toxicol 4, 305–316. doi:10.1089/aivt.2016.0026 PMC6309130 31890748

[R52] Hartung T (2018b). Making big sense from big data. Front Big Data 1, 5. doi:10.3389/fdata.2018.00005 33693321 PMC7931906

[R53] Hartung T, and Tsatsakis AM (2021). The state of the scientific revolution in toxicology. ALTEX 38, 379–386. doi:10.14573/altex.2106101 34164696

[R54] Hoffmann S and Hartung T (2005). Diagnosis: Toxic! – Trying to apply approaches of clinical diagnostics and prevalence in toxicology considerations. Toxicol Sci 85, 422–428. doi:10.1093/toxsci/kfi099 15689419

[R55] Hoffmann S, de Vries RBM, Stephens ML (2017). A primer on systematic reviews in toxicology. Arch Toxicol 91, 2551–2575. doi:10.1007/s00204-017-1980-3 28501917 PMC5489636

[R56] Hoffmann S, Kleinstreuer N, Alépée N (2018). Non-animal methods to predict skin sensitization (I): The Cosmetics Europe database. Crit Rev Toxicol 48, 344–358. doi:10.1080/10408444.2018.1429385 29474128

[R57] Hrovat M, Segner H and Jeram S (2009). Variability of in vivo fish acute toxicity data. Regul Toxicol Pharmacol 54, 294–300. doi:10.1016/j.yrtph.2009.05.013 19467285

[R58] Ioannidis JPA (2008). Effect of formal statistical significance on the credibility of observational associations. Am J Epidemiol 168, 374–383. doi:10.1093/aje/kwn156 18611956

[R59] Ioannidis JPA (2019). What have we (not) learnt from millions of scientific papers with P values? American Statistician 73, Suppl 1, 20–25. doi:10.1080/00031305.2018.1447512

[R60] IOM – Institute of Medicine (2013). Environmental Decisions in the Face of Uncertainty. Washington, DC, USA: The National Academies Press. doi:10.17226/12568 24830048

[R61] Jacobs R, van der Voet H and Braak CJFT (2015). Integrated probabilistic risk assessment for nanoparticles: The case of nanosilica in food. J Nanopart Res 17, 251. doi:10.1007/s11051-015-2911-y 26074726 PMC4457916

[R62] Jager T, den Hollander HA, Janssen GB (2000). Probabilistic risk assessment for new and existing chemicals: Example calculations. RIVM Rapport 679102049. https://www.rivm.nl/bibliotheek/rapporten/679102049.html

[R63] Jager T, Vermeire TG, Rikken MG (2001). Opportunities for a probabilistic risk assessment of chemicals in the European Union. Chemosphere 43, 257–264. doi:10.1016/s0045-6535(00)00087-4 11297405

[R64] Jaworska J, Dancik Y, Kern P (2013). Bayesian integrated testing strategy to assess skin sensitization potency: From theory to practice. J Appl Toxicol 33, 1353–1364. doi:10.1002/jat.2869 23670904

[R65] Jaworska JS, Natsch A, Ryan C (2015). Bayesian integrated testing strategy (ITS) for skin sensitization potency assessment: A decision support system for quantitative weight of evidence and adaptive testing strategy. Arch Toxicol 89, 2355–2383. doi:10.1007/s00204-015-1634-2 26612363

[R66] Jensen U (2002). Probabilistic risk analysis: Foundations and methods. J Am Stat Assoc 97, 925. doi:10.1198/016214502760301264

[R67] Judson R, Houck K, Friedman KP (2020). Selecting a minimal set of androgen receptor assays for screening chemicals. Regul Toxicol Pharmacol 117, 104764. doi:10.1016/j.yrtph.2020.104764 32798611 PMC8356084

[R68] Kafka P (1998). Observations on risk management policies, focusing on experiences from their implementation and use in the field of nuclear technology. In Proceedings of ESA Risk Management Workshop, Noordwijk (85–100). European Space Agency, ESA-ESTEC.

[R69] Kaplan S and Garrick BJ (1981). On the quantitative definition of risk. Risk Anal 1, 11–27. doi:10.1111/j.1539-6924.1981.tb01350.x 11798118

[R70] Keisler JM, Collier ZA, Chu E (2013). Value of information analysis: The state of application. Environ Syst Decis 34, 3–23. doi:10.1007/s10669-013-9439-4

[R71] Kirchsteiger C (1999). On the use of probabilistic and deterministic methods in risk analysis. J Loss Prevention Process Ind 12, 399–419. doi:10.1016/S0950-4230(99)00012-1

[R72] Kleensang A, Maertens A, Rosenberg M (2014). Pathways of toxicity. ALTEX 31, 53–61. doi:10.14573/altex.1309261 24127042 PMC4041485

[R73] Kleinstreuer NC, Browne P, Chang X (2018a). Evalua.tion of androgen assay results using a curated Hershberger database. Reprod Toxicol 81, 272–280. doi:10.1016/j.reprotox.2018.08.017 30205137 PMC7171594

[R74] Kleinstreuer NC, Hoffmann S, Alépée N (2018b). Non-animal methods to predict skin sensitization (II): An assessment of defined approaches. Crit Rev Toxicol 48, 359–374. doi:10.1080/10408444.2018.1429386 29474122 PMC7393691

[R75] Klinke A and Renn O (2002). A new approach to risk evaluation and management: Risk-based, precaution-based, and discourse-based strategies. Risk Anal 22, 1071–1094. doi:10.1111/1539-6924.00274 12530780

[R76] Krewski D, Westphal M, Andersen ME (2014). A framework for the next generation of risk science. Environ Health Perspect 122, 796–805. doi:10.1289/ehp.1307260 24727499 PMC4123023

[R77] Krewski D, Andersen M, Tyshenko MG (2020). Toxicity testing in the 21^st^ century: Progress in the past decade and future perspectives. Arch Toxicol 94, 1–58. doi:10.1007/s00204-019-02613-4 31848664

[R78] Krewski D, Saunders-Hastings P, Baan R (2022). Workshop Report: Development of an evidence-based risk assessment framwork. ALTEX, in press. 10.14573/altex.2004041 PMC10080579 36098377

[R79] Krewski D, Saunders-Hastings P, Arzuga X (in preparation). Development of a framework for evidence synthesis: Workshop report.

[R80] Kurt W (2019). Bayesian Statistics the Fun Way. No Starch Press.

[R81] Last JM (2001). A Dictionary of Epidemiology. Fourth Edition. Oxford University Press

[R82] Leist M, Hasiwa N, Rovida C (2014). Consensus report on the future of animal-free systemic toxicity testing. ALTEX 31, 341–356. doi:10.14573/altex.1406091 25061899

[R83] Leist M, Ghallab A, Graepel R (2017). Adverse outcome pathways: Opportunities, limitations and open questions. Arch Toxicol 31, 221–229. doi:10.1007/s00204-017-2045-3 29051992

[R84] Leung HW (1991). Development and utilization of physiologically based pharmacokinetic models for toxicological applications. J Toxicol Environ Health 32, 247–267. doi: 10.1080/15287399109531480 2002511

[R85] Linkov I, Massey O, Keisler J (2015). From “weight of evidence” to quantitative data integration using multicriteria decision analysis and Bayesian methods. ALTEX 32, 3–8. doi:10.14573/altex.1412231 25592482 PMC5317204

[R86] Loizou G, Spendiff M, Barton HA (2008). Development of good modelling practice for physiologically based pharmacokinetic models for use in risk assessment: The first steps. Regul Toxicol Pharmacol 50, 400–411. doi:10.1016/j.yrtph.2008.01.011 18331772

[R87] Luechtefeld T, Maertens A, McKim JM (2015). Probabilistic hazard assessment for skin sensitization potency by dose-response modeling using feature elimination instead of quantitative structure-activity relationships. J Appl Toxicol 35, 1361–1371. doi:10.1002/jat.3172 26046447 PMC4805435

[R88] Luechtefeld T, Maertens A, Russo DP (2016a). Analysis of Draize eye irritation testing and its prediction by mining publicly available 2008–2014 REACH data. ALTEX 33, 123–134. doi:10.14573/altex.1510053 26863293 PMC5461467

[R89] Luechtefeld T, Maertens A, Russo DP (2016b). Analysis of publically available skin sensitization data from REACH registrations 2008–2014. ALTEX 33, 135–148. doi:10.14573/altex.1510055 26863411 PMC5546098

[R90] Luechtefeld T, Rowlands C and Hartung T (2018a). Big-data and machine learning to revamp computational toxicology and its use in risk assessment. Toxicol Res 7, 732–744, doi:10.1039/C8TX00051D PMC6116175 30310652

[R91] Luechtefeld T, Marsh D, Rowlands C (2018b). Machine learning of toxicological big data enables read-across structure activity relationships (RASAR) outperforming animal test reproducibility. Toxicol Sci 165, 198–212. doi:10.1093/toxsci/kfy152 30007363 PMC6135638

[R92] Madsen HO, Krenk S and Lind N (1986). Method of Structure Safety. Prentice-Hall.

[R93] Maertens A, Golden E and Hartung T (2021). Avoiding regrettable substitutions: Green toxicology for sustainable chemistry. ACS Sustain Chem Eng 9, 7749–7758. doi:10.1021/acssuschemeng.0c09435 PMC9432817 36051558

[R94] McLanahan ED, El-Masri HA, Sweeney LM (2012). Physiologically based pharmacokinetic model use in risk assessment – Why being published is not enough. Toxicol Sci 126, 5–15. doi:10.1093/toxsci/kfr295 22045031

[R95] McNally K, Hogg A and Loizou G (2018). A computational workflow for probabilistic quantitative in vitro to in vivo extrapolation. Front Pharmacol 9, 441. doi:10.3389/fphar.2018.00508 29867507 PMC5968095

[R96] Meigs L, Smirnova L, Rovida C (2018). Animal testing and its alternatives – The most important omics is economics. ALTEX 35, 275–305. doi:10.14573/altex.1807041 30008008

[R97] Melchers RE (1999). Structure Reliability Analysis and Prediction. Ellis Horwood Ltd.

[R98] Mlodinow L (2008). The Drunkard’s Walk: How Randomness Rules Our Lives. Knopf Doubleday Publishing Group.

[R99] Modarres M (2008). Probabilistic risk assessment. In Misra KB (ed.), Handbook of Performability Engineering. London, UK: Springer.

[R100] Monticello TM, Jones TW, Dambach DM (2017). Current nonclinical testing paradigm enables safe entry to first-in-human clinical trials: The IQ consortium nonclinical to clinical translational database. Toxicol Appl Pharmacol 334, 100–109. doi:10.1016/j.taap.2017.09.006 28893587

[R101] Motet G and Bieder C (eds.) (2017). The Illusion of Risk Control. Springer Briefs in Applied Sciences and Technology. Cham, Switzerland: Springer.

[R102] Njå O, Solberg Ø and Braut GS (2017). Uncertainty – Its ontological status and relation to safety. In Motet G, G. and Bieder C (eds), The Illusion of Risk Control (5–21). Springer Briefs in Applied Sciences and Technology. Cham, Switzerland: Springer. doi:10.1007/978-3-319-32939-0_2

[R103] NRC – National Research Council (2007). Toxicity Testing in the 21^st^ Century: A Vision and a Strategy. Washington, DC, USA: The National Academies Press.

[R104] NRC (2009). Science and Decisions: Advancing Risk Assessment. https://www.nap.edu/read/12209/25009905

[R105] OECD (2014). The Adverse Outcome Pathway for Skin Sensitisation Initiated by Covalent Binding to Proteins. OECD Series on Testing and Assessment, No. 168. OECD Publishing, Paris. doi:10.1787/9789264221444-en

[R106] OECD (2016). Guidance Document on the Reporting of Defined Approaches to be used within Integrated Approaches to Testing and Assessment. Series on Testing and Assessment, No. 256. OECD Publishing, Paris. doi:10.1787/9789264274822-en

[R107] OECD (2018a). Test No. 442D: In Vitro Skin Sensitisation: ARE-Nrf2 Luciferase Test Method. OECD Guidelines for the Testing of Chemicals, Section 4. OECD Publishing, Paris. doi: 10.1787/9789264229822-en

[R108] OECD (2018b). Test No. 442E: In Vitro Skin Sensitisation Assays Addressing the Key Event on Activation of Dendritic Cells on the Adverse Outcome Pathway for Skin Sensitisation. OECD Guidelines for the Testing of Chemicals, Section 4. OECD Publishing, Paris. doi:10.1787/9789264264359-en

[R109] OECD (2020). Test No. 442C: In Chemico Skin Sensitisation Assays Addressing the Adverse Outcome Pathway, Key Event on Covalent Binding to Proteins. OECD Guidelines for the Testing of Chemicals, Section 4. OECD Publishing, Paris. doi: 10.1787/9789264229709-en

[R110] Olson H, Betton G, Robinson D (2000). Concordance of the toxicity of pharmaceuticals in humans and in animals. Regul Toxicol Pharmacol 32, 56–67. doi:10.1006/rtph.2000.1399 11029269

[R111] Ostrom LT and Wilhelmsen CA (2012). Probabilistic Risk Assessment. In Ostrom LT and Wilhelmsen CA (eds.), Risk Assessment (Chapter 15). John Wiley & Sons. doi:10.1002/9781118309629.ch15

[R112] Paini A, Joossens E, Bessems J (2017). EURL ECVAM Workshop on new generation of physiologically-based kinetic models in risk assessment. European Union, 2017. JRC108231, EUR 28794 EN doi:10.2760/619902

[R113] Pardo O, Beser MI, Yusà V (2014). Probabilistic risk assessment of the exposure to polybrominated diphenyl ethers via fish and seafood consumption in the region of Valencia (Spain). Chemosphere 104, 7–14. doi:10.1016/j.chemosphere.2013.12.084 24534151

[R114] Pariès J (2017). Recognizing complexity in risk management: The challenge of the improbable. In Motet G and Bieder C (eds.), The Illusion of Risk Control (41–55). Springer Briefs in Applied Sciences and Technology. Cham, Switzerland: Springer. doi:10.1007/978-3-319-32939-0_4

[R115] Parkin RT and Morgan MG (2006). Examples of potential benefits of probabilistic risk analysis. Cover letter and attachment to Stephen L. Johnson, Administrator, U.S. Environmental Protection Agency, from the EPA Science Advisory Board, Washington, DC, December 6.

[R116] Partosch F, Mielke H and Stahlmann R (2015). Internal threshold of toxicological concern values: Enabling route-to-route extrapolation. Arch Toxicol 89, 941–948. doi:10.1007/s00204-014-1287-6 24915937

[R117] Pearl J (1988). Probabilistic Reasoning in Intelligent Systems: Networks of Plausible Inference. Representation and Reasoning Series (2^nd^ printing edition). San Francisco, California, USA: Morgan Kaufmann. doi:10.1016/C2009-0-27609-4

[R118] Pendse S, Clewell R, Efremenko A (2017). PLETHEM–An interactive open-source platform for bridging the source-to-outcome continuum. Toxicol Lett 280, S288. doi:10.1016/j.toxlet.2017.07.807

[R119] Rosling H, Rosling Rönnlund A and Rosling O (2018). Factfulness: Ten Reasons We’re Wrong About the World – And Why Things Are Better Than You Think. New York, USA: Flatiron Books, Macmillan Publishers.

[R120] Rossi RJ (2018). Mathematical Statistics: An Introduction to Likelihood Based Inference (p. 227). New York; USA: John Wiley & Sons.

[R121] Rovida C, Alépée N, Api AM (2015). Integrated testing strategies (ITS) for safety assessment. ALTEX 32, 171–181. doi:10.14573/altex.1506201 25413849

[R122] Rovida C, Barton-Maclaren T, Benfenati E (2020). Internationalisation of read-across as a validated new approach method (NAM) for regulatory toxicology. ALTEX 37, 579–606. doi:10.14573/altex.1912181 32369604 PMC9201788

[R123] Rysavy M (2013). Evidence-based medicine: A science of uncertainty and an art of probability. Virtual Mentor 15, 4–8. doi:10.1001/virtualmentor.2013.15.1.fred1-1301 23356799

[R124] Sala Benito JV, Paini A, Richarz AN (2017). Automated workflows for modelling chemical fate, kinetics and toxicity. Toxicol In Vitro 45, 249–257. doi:10.1016/j.tiv.2017.03.004 28323105 PMC5745146

[R125] Saltelli A, Ratto M, Andres T (2008). Global Sensitivity Analysis. The Primer. Chichester, UK: John Wiley & Sons Ltd, The Atrium, Southern Gate. https://bit.ly/3q69Q6O

[R126] Sanderson H (2003). Probabilistic hazard assessment of environmentally occurring pharmaceuticals toxicity to fish, daphnids and algae by ECOSAR screening. Toxicol Lett 144, 383–395. doi:10.1016/s0378-4274(03)00257-1 12927355

[R127] Santín EP, Solana RR, García MG (2021). Toxicity prediction based on artificial intelligence: A multidisciplinary overview. Wiley Interdiscip Rev Comput Mol Sci, e1516. doi:10.1002/wcms.1516

[R128] Scheringer M, Steinbach D, Escher B (2002). Probabilistic approaches in the effect assessment of toxic chemicals – What are the benefits and limitations? Environ Sci Pollut Res Int 9, 307–314. doi:10.1065/espr2001.09.091 12391805

[R129] Schleier JJ, Marshall LA, Davis RS (2015). A quantitative approach for integrating multiple lines of evidence for the evaluation of environmental health risks. PeerJ 3, e730. doi: 10.7717/peerj.730 25648367 PMC4304847

[R130] Schowanek D, David H, Francaviglia R (2007). Probabilistic risk assessment for linear alkylbenzene sulfonate (LAS) in sewage sludge used on agricultural soil. Regul Toxicol Pharmacol 49, 245–259. doi:10.1016/j.yrtph.2007.09.001 17967498

[R131] Shah I, Liu J, Judson RS (2016). Systematically evaluating read-across prediction and performance using a local validity approach characterized by chemical structure and bioactivity information. Regul Toxicol Pharmacol 79, 12–24. doi:10.1016/j.yrtph.2016.05.008 27174420

[R132] Sillé FCM, Karakitsios S, Kleensang A (2020). The exposome – A new approach for risk assessment. ALTEX 37, 3–23. doi:10.14573/altex.2001051 31960937 PMC13041581

[R133] Slob W, Bakker MI, te Biesebeek JD (2014). Exploring the uncertainties in cancer risk assessment using the integrated probabilistic risk assessment (IPRA) approach. Risk Anal 34, 1401–1422. doi:10.1111/risa.12194 24766324

[R134] Smirnova L, Kleinstreuer N, Corvi R (2018). 3S – Systematic, systemic, and systems biology and toxicology. ALTEX 35, 139–162. doi:10.14573/altex.1804051 29677694 PMC6696989

[R135] Solomon K, Giesy J and Jones P (2000). Probabilistic risk assessment of agrochemicals in the environment. Crop Protection 19, 649–655. doi:10.1016/S0261-2194(00)00086-7

[R136] Sterne JA, Hernán MA, Reeves BC (2016). ROBINS-I: A tool for assessing risk of bias in non-randomised studies of interventions. BMJ 355, i4919. doi:10.1136/bmj.i4919 27733354 PMC5062054

[R137] Szucs D and Ioannidis JPA (2017). When null hypothesis significance testing is unsuitable for research: a reassessment. Front Hum Neurosci 11, 390. doi:10.3389/fnhum.2017.00390 28824397 PMC5540883

[R138] Taleb NN (2004). Fooled by Randomness – The Hidden Role of Chance in Life and the Markets. London, UK: Penguin. (2^nd^ edition).

[R139] Taleb NN (2007). The Black Swan – The Impact of the Highly Improbable. New York, USA: The Random House Publishing Group.

[R140] Tollefsen KE, Scholz S, Cronin MT (2014). Applying adverse outcome pathways (AOPs) to support integrated approaches to testing and assessment (IATA). Regul Toxicol Pharmacol 70, 629–640. doi:10.1016/j.yrtph.2014.09.009 25261300

[R141] Tomasetti C, Li L and Vogelstein B (2017). Stem cell divisions, somatic mutations, cancer etiology, and cancer prevention. Science 355, 1330–1334. doi:10.1126/science.aaf9011 28336671 PMC5852673

[R142] Tralau T, Oelgeschläger M, Gürtler R (2015). Regulatory toxicology in the twenty-first century: Challenges, perspectives and possible solutions. Arch Toxicol 89, 823–850. doi:10.1007/s00204-015-1510-0 25820917

[R143] Tsaioun K, Blaauboer BJ and Hartung T (2016). Evidence-based absorption, distribution, metabolism, excretion and toxicity (ADMET) and the role of alternative methods. ALTEX 33, 343–358. doi:10.14573/altex.1610101 27806179

[R144] Tsaioun K (in preparation). Advancing the application of evidence-based methods to construct mechanistic frameworks for the development and use of non-animal toxicity tests.

[R145] van der Voet H and Slob W, (2007). Integration of probabilistic exposure assessment and probabilistic hazard characterization. Risk Anal 27, 351–371. doi:10.1111/j.1539-6924.2007.00887.x 17511703

[R146] Verdonck FAM, Jaworska J, Janssen CR (2002). Probabilistic ecological risk assessment framework for chemical substances. In Integrated Assessment and Decision Support. Proceedings of the First Biennial Meeting of the International Environmental Modelling and Software Society (IEMSS). https://former.iemss.org/sites/iemss2002/proceedings/pdf/volume%20uno/24_verdonck.pdf

[R147] Verdonck FAM, Aldenberg T, Jaworska J (2003). Limitations of current risk characterization methods in probabilistic environmental risk assessment. Environ Toxicol Chem 22, 2209–2213. doi:10.1897/02-435 12959553

[R148] Vesely WE (2011). Probabilistic Risk Assessment. In Johnson SB, Gormley TJ, Kessler SS (eds.), System Health Management: With Aerospace Applications (Chapter 15). John Wiley & Sons. doi:10.1002/9781119994053.ch15

[R149] Vinken M, Benfenati E, Busquet F (2021). Safer chemicals using less animals: Kick-off of the European ONTOX project. Toxicology 458, 152846. doi:10.1016/j.tox.2021.152846 34216698

[R150] Vlek C (2010). Judicious management of uncertain risks: I. Criticisms and developments of risk analysis and precautionary reasoning. II. Simple rules and more intricate models for precautionary decision-making. J Risk Res 13, 517–543. doi:10.1080/13669871003629887

[R151] Vose D (2008). Risk Analysis: A Quantitative Guide. 3^rd^ edition. Chichester, UK: John Wiley & Sons.

[R152] Wagner C, Zhao P, Pan Y (2015). Application of physiologically based pharmacokinetic (PBPK) modeling to support dose selection: Report of an FDA public workshop on PBPK. CPT: Pharmacometrics Syst Pharmacol 4, 226–230. doi:10.1002/psp4.33 26225246 PMC4429576

[R153] Walker WE, Harremoës P, Rotmans J (2003). Defining uncertainty, a conceptual basis for uncertainty management in model-based decision support. Integr Assess 4, 5–17. 10.1076/iaij.4.1.5.16466

[R154] Wambaugh JF, Wetmore BA, Pearce R (2015). Toxicokinetic triage for environmental chemicals. Toxicol Sci 147, 55–67. doi:10.1093/toxsci/kfv118 26085347 PMC4560038

[R155] Wang B and Gray G (2015). Concordance of noncarcinogenic endpoints in rodent chemical bioassays. Risk Anal 35, 1154–1166. doi:10.1111/risa.12314 25545328

[R156] Wang Y-H (2010). Confidence assessment of the Simcyp time-based approach and a static mathematical model in predicting clinical drug-drug interactions for mechanism-based CYP3A inhibitors. Drug Metab Dispos 38, 1094–1104. doi:10.1124/dmd.110.032177 20368327

[R157] Wheelan C (2013). Naked Statistics: Stripping the Dread from the Data. W.W. Norton & Company.

[R158] Xie J, Marano KM, Wilson CL (2012). A probabilistic risk assessment approach used to prioritize chemical constituents in mainstream smoke of cigarettes sold in China. Regul Toxicol Pharmacol 62, 355–362. doi:10.1016/j.yrtph.2011.10.017 22085590

[R159] Young B, Tulve N, Egeghy P (2012). Comparison of four probabilistic models (CARES^®^, Calendex^™^, ConsExpo, and SHEDS) to estimate aggregate residential exposures to pesticides. J Expo Sci Environ Epidemiol 22, 522–532. doi:10.1038/jes.2012.54 22781436

[R160] Zhang L, Zhang H, Ai H (2018). Applications of machine learning methods in drug toxicity prediction. Curr Top Med Chem 18, 987–997. doi:10.2174/1568026618666180727152557 30051792

[R161] Zhang P (2010). Probabilistic methods used in environmental risk evaluation for groundwater protection. Dissertation, Faculty of Mathematics and Natural Sciences Department of Geosciences, University of Oslo. https://www.duo.uio.no/bitstream/handle/10852/12317/Zhang-avhandling-publ.pdf?sequence=3&isAllowed=y

[R162] Zhao F, Li L, Chen Y, Huang Y (2021). Risk-based chemical ranking and generating a prioritized human exposome database. Environ Health Perspect 129, 47014. doi:10.1289/ehp7722 33929905 PMC8086799

[R163] Zhu H, Bouhifd M, Kleinstreuer N (2016). Supporting read-across using biological data. ALTEX 33, 167–182. doi:10.14573/altex.1601252. 26863516 PMC4834201

